# A Review of MAX Series Materials: From Diversity, Synthesis, Prediction, Properties Oriented to Functions

**DOI:** 10.1007/s40820-025-01673-9

**Published:** 2025-03-03

**Authors:** Jian Zhang, Ru Jia, Kar Ban Tan, Jiaming Li, Shichong Xu, Guobing Ying, Wenjuan Han, Ming Lu

**Affiliations:** 1 The Joint Laboratory of MAX/MXene Materials, Key Laboratory of Functional Materials Physics and Chemistry of the Ministry of Education, Jilin Normal University, Changchun, 130103 People’s Republic of China; 2 School of Materials Science and Engineering, Southeast University, Nanjing, 211189 People’s Republic of China; 3 Department of Chemistry, Faculty of Science, Universiti Putra Malaysia, 43400 Serdang, Malaysia; 4 Shenyang National Laboratory for Materials Science, Institute of Metal Research, Chinese Academy of Sciences, Shenyang, 110016 People’s Republic of China

**Keywords:** MAX materials, Diversity, Synthetic strategy, Prediction, Function

## Abstract

MAX series materials, as non-van der Waals layered multi-element compounds, contribute remarkable regulated properties and functional dimension, combining the features of metal and ceramic materials due to their inherently laminated crystal structure that M_n+1_X_n_ slabs are intercalated with A element layers. Oriented to the functional requirements of information, intelligence, electrification, and aerospace in the new era, how to accelerate MAX series materials into new quality productive forces? The systematic enhancement of knowledge about MAX series materials is intrinsic to understanding its low-dimensional geometric structure characteristics, and physical and chemical properties, revealing the correlation of composition, structure, and function and further realizing rational design based on simulation and prediction. Diversity also brings complexity to MAX materials research. This review provides substantial tabular information on (I) MAX’s research timeline from 1960 to the present, (II) structure diversity and classification convention, (III) synthesis route exploration, (IV) prediction based on theory and machine learning, (V) properties, and (VI) functional applications. Herein, the researchers can quickly locate research content and recognize connections and differences of MAX series materials. In addition, the research challenges for the future development of MAX series materials are highlighted.

## Highlights

- Oriented to the understanding of MAX series materials, the research timeline, structure diversity, and synthesis are systematically reviewed.
- The prediction, properties, and functional applications of MAX series materials are summarized.
- This review emphasizes research challenges for the future development of MAX series materials.

## Graphical abstract

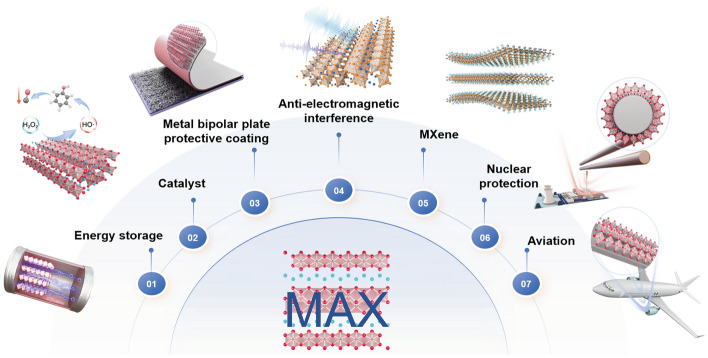

## Introduction

**Fig. 1:**
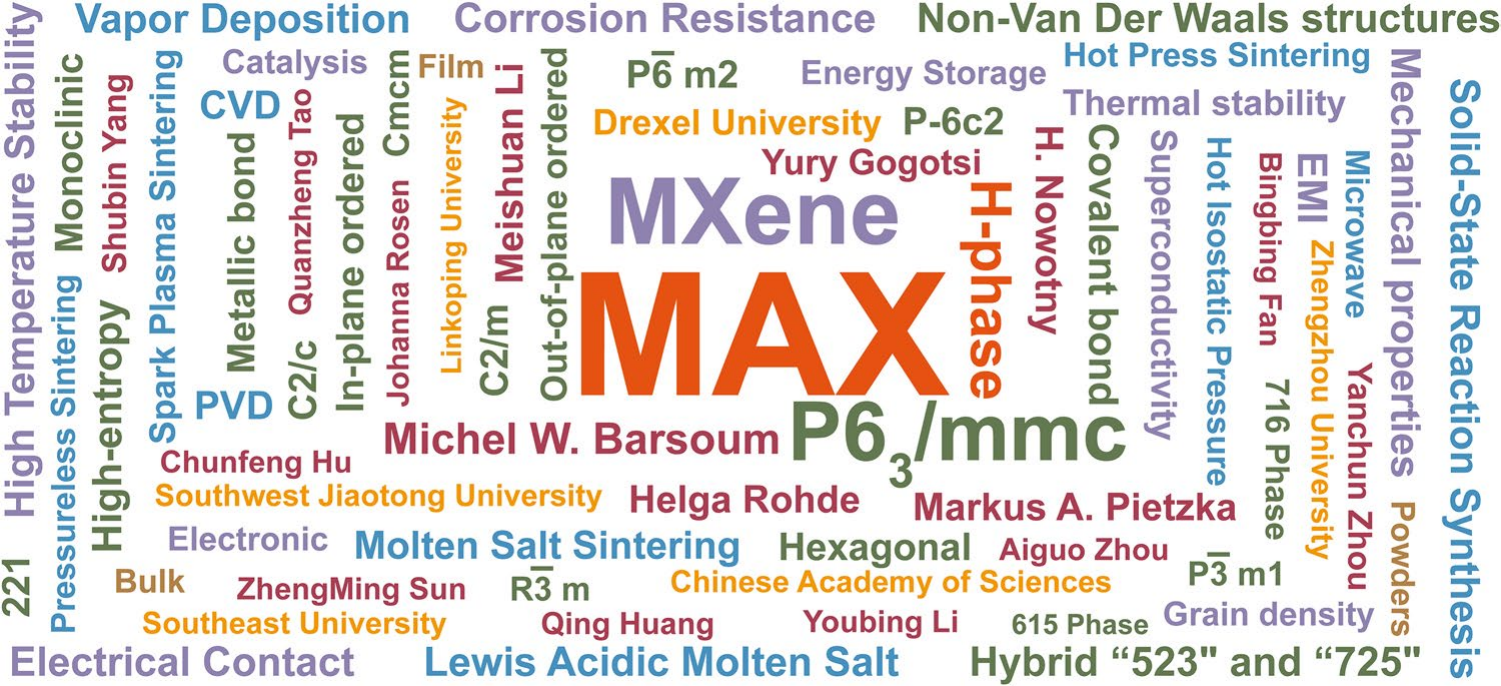
High-frequency keywords of MAX series materials

Oriented to the functional requirements of information, intelligence, electrification, and aerospace in new era, materials science research is the cornerstone of supporting technological innovation, which can endow equipment and systems with new functions and characteristics in various fields, accelerating the realization of technological breakthroughs. Due to their similar atomic arrangements, a series of transition metal carbides, nitrides, and carbonitrides are categorized as MAX series materials (MAXs), once named H-phases in 1960s [1]. Up to now, over 383 different types of MAXs have been reported based on synthetic strategy innovation on solid-state reaction sintering, melting reaction, and physicochemical deposition. Meanwhile, a series of novel MAXs are predicted by theoretical simulation and machine learning. The diversity investigations in elemental composition and structure bring the adjustable properties: ceramic characteristics (high-temperature resilience [2], strength [3], and oxidation resistance [4]); metallic properties (conductivity [5], thermal conduction [6], machinability [7], and impact durability [8]). MAXs are intended in the potential function requirements in rail transportation lubrication [9], heating components [10], electrical contacts [11, 12], electromagnetic shielding [13], microwave absorption [14], high-level radioactive waste solidification [15], and electrochemical energy storage [16, 17]. MXene series materials, as the low-dimensional derivatives, showed potential applications in electrochemical energy storage [18], luminescence [19], catalysis, and other fields [20, 21]. Figure 1 shows the high-frequency keywords of MAX’s research. However, MAXs are not a material cornerstone to future industrialization prospects. How to accelerate MAXs into new quality productive forces? It is intrinsic to understand its low-dimensional geometric structure characteristics, and physical and chemical properties, to reveal the correlation of composition, structure, and function and further to realize rational design based on simulation and prediction.

Herein, oriented toward structure and function correlation, the information retrieval on (I) MAX’s research timeline from 1960 to the present, (II) structure diversity and classification convention, (III) synthesis route exploration, (IV) prediction based on theory and machine learning, (V) properties, and (VI) functional applications are described in categories to help readers quickly understand the research progress of MAXs. Moreover, by integrating advanced synthesis and characterization techniques and machine learning, some existing problems are addressed, and future research directions are prospected.

## Historic Milestones and Timeline

**Fig. 2:**
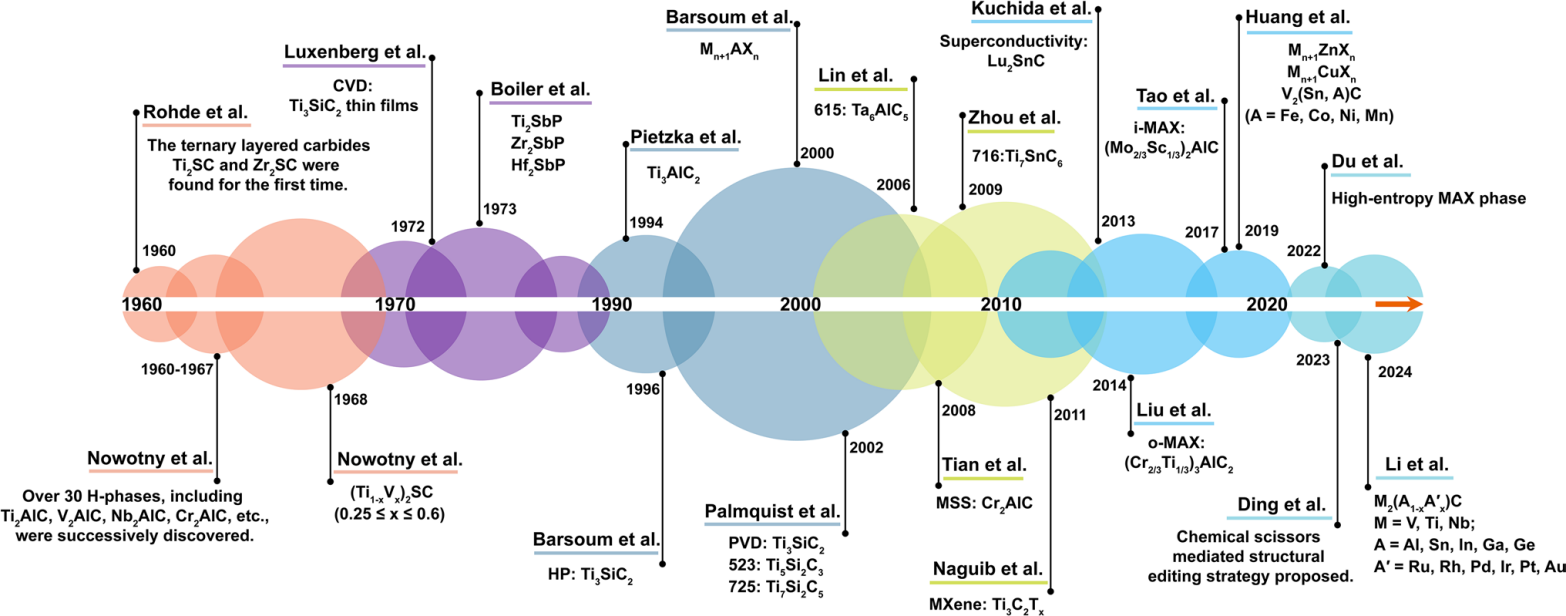
Timeline of MAX series materials

Reviewing the history of MAXs helps understand the limitations of science, technology, and society on the innovative research, as shown in Fig. 2. Back to 1960, Rohde et al. [22] found Ti_4_S_2_C_2_ and Zr_4_S_2_C_2_ by heat treatment of Ti, S, C, and Zr at 1600 °C. Between 1960 and 1967, Nowotny et al. [23–26] synthesized a series of ternary layered carbides/nitrides, including Ti_2_AlC, V_2_AlC, Cr_2_AlC, and Nb_2_AlC, which were named as H-phases. In 1970s, Nickl et al. [27] prepared Ti_3_SiC_2_ by chemical vapor deposition (CVD). In 1994, Pietzka et al. [28] synthesized Ti_3_AlC_2_ by a cold pressing sintering method and proposed the thermochemical stability limitation based on the formation free energy of the binary intermediate phases of TiAl, TiC, and AlC. In 1996, Prof. Barsoum et al. [8] achieved a dense Ti_3_SiC_2_ MAX bulk by reactive hot pressing (HP) technology. In 2000s, a review article entitled "The M_N+1_AX_N_ Phases: A New Class of Solids; Thermodynamically Stable Nanolaminates" was published in Prog. Solid St. Chem. The concept of "M_n+1_AX_n_ phases (MAX)" was proposed based on the unique structural features and properties, which opened a new era of MAXs [1].

In 2002, Palmquist et al. [29] employed DC magnetron sputtering technique to prepare the oriented Ti_3_SiC_2_ and Ti_4_SiC_3_ MAX single-crystal thin films; in addition, two previously unknown compounds of Ti_5_Si_2_C_3_ and Ti_7_Si_2_C_5_ MAXs were observed. In 2006, Lin et al. [30] found a previously unknown Ta_6_AlC_5_ in the ternary Ta-Al-C system. In 2008, Tian et al. [31] prepared high-purity Cr_2_AlC using molten salt sintering, which reduce the sintering temperature by 200 °C. This is a breakthrough in the MAX preparation strategy. In 2009, Zhang et al. [32] determined a new MAX phase (716-phase Ti_7_SnC_6_). In 2011, Naguib et al. [33] found "MXene," "MX" stands for the element left after MAX etching, and "ene" stands for the 2D material structure features. In 2014, Liu et al. [34] reported the first out-of-plane ordered MAX phase exhibiting perpendicular to the M-layer, called o-MAX. In 2017, another type of ordered MAX called in-plane ordered MAX (i-MAX) was first discovered by Tao et al.[35]. In 2019, Huang et al. [36, 37] used Lewis acid molten salt to realize the element replacement and created a series of new MAXs containing Zn and Cu elements at A-sites. In 2019, Li et al. [38] synthesized V_2_(A_*x*_Sn_1−*x*_)C MAX (A = Fe, Co, Ni, Mn or their binary/ternary/quadratic combinations) based on alloying-guided reactions. In 2022, by pressureless sintering at 1500 °C, Du et al. [39] developed a series of high-entropy MAXs and further the high-entropy MXene. In 2023, Ding et al. [40] proposed a chemical scissor-mediated structural editing strategy to allow the unconventional elements into interlayer atom vacancies to form new MAXs, thus revolutionizing traditional metallurgic reactions. In 2024, Li et al. [41] reported a universal method of A-site preferential alloying to form noble metal MAXs.

Thanks to the fine structural analysis of the MAXs by early researchers, this is the foundation for discovering structural similarities. Contributions to the development of preparation methods allow us to see the diversity of MAXs. Upon application requirements, the chemical and physical properties, as well as the functional applications, are investigated. In the past 60 years, progress in basic research of MAXs comes alongside successes in preparation, characterization, property, and function.

## Diversity and Classification Convention

### Element Diversity

**Fig. 3:**
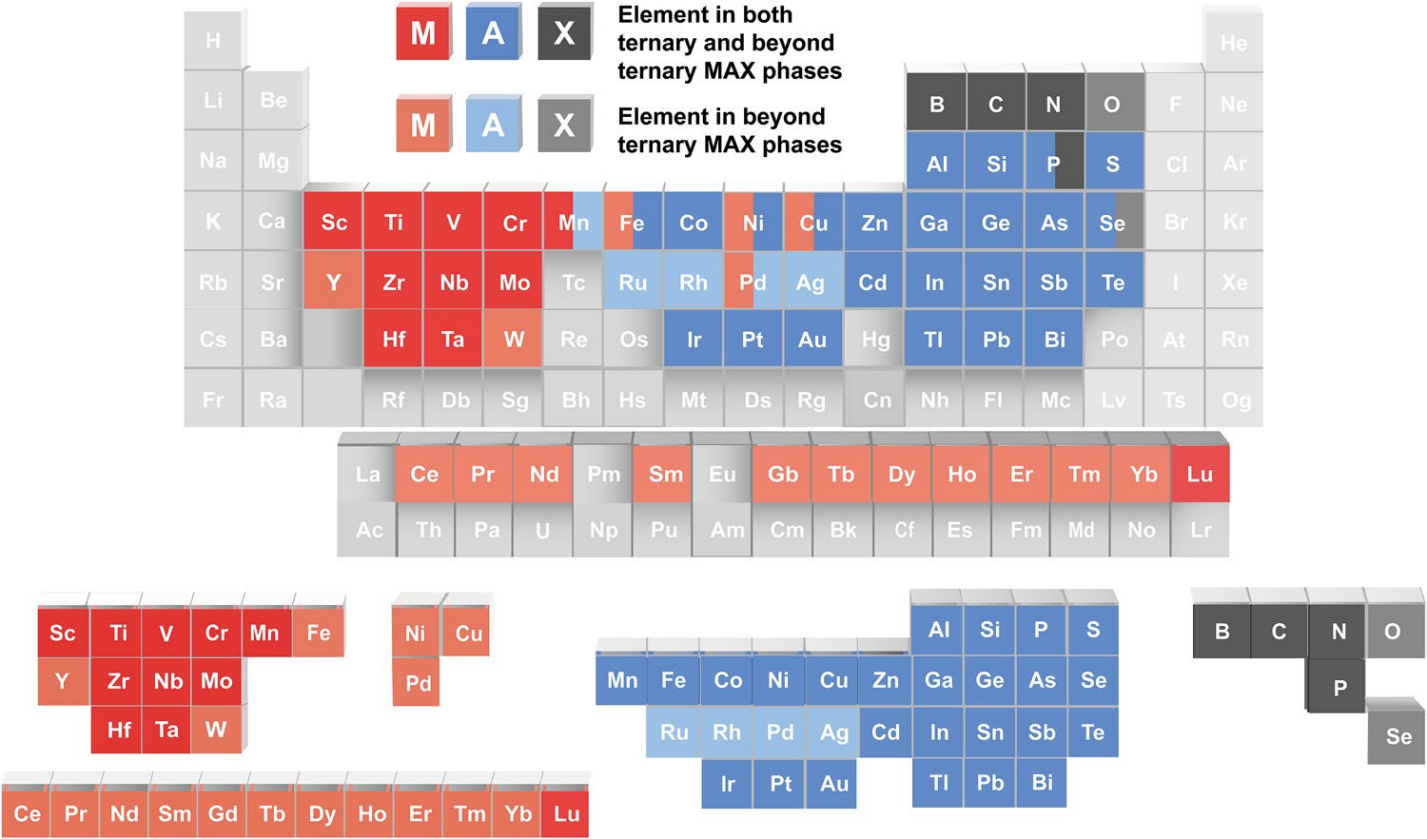
Periodic table of the elements in the MAX series materials

In the up-to-date periodic table of the elements, 28 M, 29 A, and 6 X-site elements are found that can be utilized to form MAXs. This means that MAXs can contain nearly 50% of elements, as shown in Fig. 3. So why do MAX show such strong elemental inclusiveness? This is due to the unique layered structure, as well as the bonding and arrangement between M-A and M-X, which gives the atoms a high freedom degree of spatial and chemical coordination in their arrangement and bonding. Of course, the reported elemental composition also reflects the rules.

In the M-site, there are 28 kinds of elements that can participate in the composition, and the elements in the M-site have been extended from the previously well-known transition metallic elements, such as Ti, V, and Cr [23–25], to the rare-earth elements, such as Ce, Pr, and Nd [42–44]. Among them, lanthanide elements can participate in the M-site with MM' as an ordered solid solution state. Fe, Ni, Cu, and Pd can only exist in solid solution at the M-site with other elements [45–47]. The element of W can participate in both ordered and disordered solid solutions but cannot exist at the M-site alone [44, 48]. Hf, Ta can appear in M-site disordered MAX [49, 50]. Mn, Zr, Sc, and Y have been added to the M-site element [51–53]. Meanwhile, a series of i-MAXs containing Ce, Pr, Nd, Sm, Gd, Tb, Dy, Ho, Er, Tm, and Lu also are introduced in the M-sites [44].

For A-site, there are 29 kinds of elements that can participate in the composition, including group IIIA, group IVA, and transition metal elements such as Au, Ir, Zn, Cu, Fe, Co, Ni, Sb, and Pt [40, 41, 54]. In addition, P, S, As, Te, and Tl are reported to participate in the formation of ternary A-site [23, 55–57]. Mn, Rh, Pd, and Ag are reported to appear at A-site with other elements as solid solutions [38, 40, 41]. Au, Ir, and Zn are introduced by substitution reaction at the A-site [36, 54]. These magnetic elements of Fe, Co, Ni, and Mn were utilized to prepare V_2_(A_*x*_Sn_1−*x*_)C [38]. Fe was introduced to form Ta_2_FeC, Ti_2_FeN, and Nb_2_FeC [58]. Relying on a chemical structure editing strategy, the unconventional elements (Bi, Sb, Fe, etc.) can be intercalated into A-sites [40]. A series of noble metal elements were introduced to prepare M_2_(A_1−*x*_A′_*x*_)C (where M = Ti, V, or Nb; A = Sn, Al, Ge, Ga, and In; and A′ = Ru, Rh, Pd, Pt, Ir, and Au, with 0 < *x* ≤ 0.4) by the method of A-site alloying-guided strategy [41].

X-sites include C, N, B, P, O, and Se. C, N, and B can exist independently. Relying on the partial substitution strategy of X, Ti_2_AlC_1−*x*_O_*x*_, Nb_2_SB_*x*_C_1−*x*_, Zr_2_Se(B_1−*x*_Se_*x*_) show that elements O and Se can only be combined with C and B at X-site [59–61]. The B-containing MAXs with a symmetry of P6_3_/mmc are different from MAB materials [62–64]. In addition, Hf_2_SB, Ti_2_SbP, Zr_2_SbP, and Hf_2_SbP containing P MAXs are reported.

Based on the combination of different elements, MAXs show great element compatibility. The inherent features of different elements induced the diversity of MAX in structure, properties, and functions. The element combination rules are essential for expanding the types of MAXs. To facilitate the search, the reported MAXs are classified in Table 1.

**Table 1:** Reported MAX series materials

211 Phase									
Ti_2_AlC [24]	Ti_2_AlN [65]	V_2_AlC [66]	Cr_2_AlC [67]	Zr_2_AlC [68]	Nb_2_AlC [69]	Ta_2_AlC [70]	Hf_2_AlC [71]	V_2_PC [72]	Nb_2_PC [57]
Sc_2_SC *	Sc_2_SP *	Ti_2_SC [22]	Y_2_SC*	Zr_2_SB [73]	Zr_2_SC [22]	Zr_2_SN *	Zr_2_SP *	Nb_2_SB [63]	Nb_2_SC [57]
Hf_2_SB [73]	Hf_2_SC [74]	Ti_2_FeN [58]	Nb_2_FeC [58]	Ta_2_FeC [58]	Nb_2_CoC [40]	Ta_2_CoC[75]	Nb_2_NiC [40]	Ta_2_NiC [75]	Nb_2_CuC [76]
Ti_2_ZnC [36]	Ti_2_ZnN [36]	V_2_ZnC [36]	Mn_2_ZnC *	Mn_2_ZnN *	Nb_2_ZnC [40]	Ti_2_GaC [77]	Ti_2_GaN [77]	V_2_GaC [78]	V_2_GaN [78]
Cr_2_GaC [25]	Cr_2_GaN [79]	Mn_2_GaC [80]	Nb_2_GaC [77]	Mo_2_GaC [24]	Hf_2_GaC *	Hf_2_GaN *	Ta_2_GaC [81]	Ti_2_GeC [26]	Ti_2_GeN *
V_2_GeC [25]	V_2_GeN [82]	Cr_2_GeC [25]	Zr_2_GeC [83]	Nb_2_GeC [84]	Ta_2_GeC *	Ti_2_AsC *	Ti_2_AsP *	V_2_AsC [56]	Zr_2_AsP *
Nb_2_AsC [57]	Hf_2_AsP *	Ta_2_AsC *	Ta_2_AsB *	Sc_2_SeC *	Sc_2_SeP *	Ti_2_SeC *	Ti_2_SeP *	Y_2_SeC *	Y_2_SeP *
Zr_2_SeB [85]	Zr_2_SeC [6]	Zr_2_SeP *	Hf_2_SeB [85]	Hf_2_SeC [86]	V_2_PdC *	Sc_2_CdC *	Ti_2_CdC [77]	Ti_2_CdN *	Zr_2_CdC *
Zr_2_CdN *	Hf_2_CdC*	Hf_2_CdN *	Ti_2_InC [26]	Ti_2_InN [77]	Zr_2_InC [26]	Zr_2_InN [77]	Zr_2_InP *	Nb_2_InC [81]	Hf_2_InB *
Hf_2_InC [26]	Hf_2_InN [82]	Sc_2_SnC [87]	Sc_2_SnP *	Ti_2_SnC [88]	Ti_2_SnN*	V_2_SnC [89]	Zr_2_SnC [88]	Nb_2_SnC [88]	Nb_2_SnB [83]
Hf_2_SnC [88]	Hf_2_SnN [90]	Lu_2_SnC [5]	Sc_2_SbC *	Ti_2_SbC *	Ti_2_SbP [91]	Zr_2_SbP [91]	Nb_2_SbC [40]	Hf_2_SbP [91]	Sc_2_TeC *
Sc_2_TeP *	Y_2_TeC *	Y_2_TeP *	Zr_2_TeC *	Zr_2_TeP *	Hf_2_TeB [55]	Hf_2_TeC *	V_2_IrC *	Mn_2_IrN *	Nb_2_PtC [40]
V_2_PtC *	Cr_2_PtC *	V_2_AuC *	Nb_2_AuC [40]	Mo_2_AuC [92]	Sc_2_HgC *	Ti_2_HgC	Ti_2_HgN *	Zr_2_HgC	Zr_2_HgN *
Hf_2_HgC *	Hf_2_HgN *	Sc_2_TlN *	Sc_2_TlP *	Ti_2_TlC [81]	Zr_2_TlC [23]	Zr_2_TlN [82]	Zr_2_TlP *	Hf_2_TlC [23]	Sc_2_PbC [93]
Sc_2_PbN *	Sc_2_PbP *	Ti_2_PbC [81]	Y_2_PbC *	Y_2_PbP *	Zr_2_PbB *	Zr_2_PbC [23]	Zr_2_PbN *	Zr_2_PbP *	Hf_2_PbB *
Hf_2_PbC [23]	Sc_2_BiC *	Ti_2_BiC *	Zr_2_BiC *	Zr_2_BiP *	Hf_2_BiB *	Hf_2_BiC *	Hf_2_BiP *		

211 Phase: M-site solid solution			
(Ti_*x*_V_1−*x*_)_2_AlC (*x* = 0.5, 1, 1.5)[94]	(Ti_*x*_Cr_1−*x*_)_2_AlC(*x* = 0.5, 1.5) [94]	(Ti, Zr)_2_AlC [95]	(Ti_1−*x*_Nb_*x*_)_2_AlC [96]
(Ti_1−*x*_Mo_*x*_)_2_AlC (0 < *x* < 0.2) [97]	(Ti_1−*x*_Ta_*x*_)_2_AlC(0 < *x* < 1) [98]	(Ti_0.1_V_0.8_Cr_0.1_)_2_AlC [99]	(TiVNb)_2_AlC [100]
(Ti, Nb, Ta)_2_AlC [39]	(Ti, V, Nb, Ta)_2_AlC [101]	(Ti, Zr, Nb, Ta)_2_AlC [39]	(Ti_0.27_Zr_0.21_Nb_0.24_Ta_0.28_)_2_AlC [102]
(Ti_1/5_V_1/5_Cr_1/5_Nb_1/5_Ta_1/5_)_2_AlC [103]	(TiHfVNbTa)_2_AlC [101]	(Ti, Zr, Hf, Nb, Ta)_2_AlC [104]	(Ti_1/5_V_1/5_Zr_1/5_Nb_1/5_Ta_1/5_)_2_AlC [39]
(V_*x*_Cr_1−*x*_)_2_AlC (*x* = 0.5, 1, 1.5) [94]	(V_0.96_Mn_0.04_)_2_AlC [105]	(V_*x*_Nb_1−*x*_)_2_AlC [106]	(V_1−*x*_Ta_*x*_)_2_AlC [107]
(Cr_0.8_Mn_0.2_)_2_AlC [108]	(Cr_1−*x*_Mn_*x*_)_2_AlC(x < 0.1) [109]	(Cr_1−*x*_Fe_*x*_)_2_AlC(*x* < 0.02) [109]	(Nb_2/3_Sc_1/3_)_2_AlC [110]
(Nb_1−*x*_Zr_*x*_)_2_AlC [106]	(Nb_1−*x*_Ta_*x*_)_2_AlC [111]	(Hf_1−*x*_Ta_*x*_)_2_AlC [111]	(Ti_1−*x*_V_*x*_)_2_SC (0.25 ≤ *x* ≤ 0.6) [57]
(Ti, Zr, Hf)_2_SC [53]	(Ti_0.33_Nb_0.33_Ta_0.33_)_2_FeC [49]	(Ti_0.2_Zr_0.2_V_0.2_Nb_0.2_Ta_0.2_)_2_FeC [49]	(V_1-x_Cr_x_)_2_GaC(0 < x < 1) [112]
(Cr_0.5_Mn_0.5_)_2_GaC [113]	(Cr_2−*x*_Mn_*x*_)GaC (0 ≤ *x* ≤ 1) [114]	(Mo_0.5_Mn_0.5_)_2_GaC [115]	(Ti,V, Nb)_2_GaC [116]
(Ti, V, Nb, Ta)_2_GaC [116]	(Ti, V, Nb, Ta, Mo)_2_GaC [116]	(Ti_1−*x*_V_*x*_)_2_GeC (*x* ≈ 0.5) [117]	Cr_2−*x*_Ti_*x*_GeC (*x* ≥ 0.75) [118]
Cr_2−*x*_V_*x*_GeC [118]	Cr_2−*x*_Mn_*x*_GeC [118]	Cr_2−*x*_Fe_*x*_GeC (*x* ≤ 0.1) [118]	Cr_2−*x*_Mo_*x*2_GeC (*x* ≤ 0.5) [118]
(Ti, Zr)_2_InC [119]	(Ti, Hf)_2_InC [120]	(TiVNb)_2_SnC [50]	(TiVNbZr)_2_SnC [50]
(Ti, V, Nb, Zr, Hf)_2_SnC [50]	(Cr_0.5_Mn_0.5_)_2_AuC [51]		

211 Phase: i-MAX					
(Sc_2/3_W_1/3_)_2_AlC *	(Ti_2/3_Zr_1/3_)_2_AlC *	(Ti_2/3_Y_1/3_)_2_AlC *	(V_2/3_Sc_1/3_)_2_AlC [121]	(V_2/3_Y_1/3_)_2_AlC *	(V_2/3_Zr_1/3_)_2_AlC [122]
(V_2/3_Hf_1/3_)_2_AlC *	(Cr_2/3_Sc_1/3_)_2_AlC [52]	(Cr_2/3_Y_1/3_)_2_AlC [52]	(Cr_2/3_Zr_1/3_)_2_AlC [123]	(Cr_2/3_Nb_1/3_)_2_AlC *	(Cr_2/3_Hf_1/3_)_2_AlC *
(Cr_2/3_Ta_1/3_)_2_AlC *	(Cr_2/3_Gd_1/3_)_2_AlC [124]	(Cr_2/3_Tb_1/3_)_2_AlC [124]	(Cr_2/3_Dy_1/3_)_2_AlC [124]	(Cr_2/3_Ho_1/3_)_2_AlC [124]	(Cr_2/3_Er_1/3_)_2_AlC [124]
(Cr_2/3_Tm_1/3_)_2_AlC [124]	(Cr_2/3_Lu_1/3_)_2_AlC [124]	(Mn_2/3_Sc_1/3_)_2_AlC *	(Mn_2/3_Y_1/3_)_2_AlC *	(Mn_2/3_Zr_1/3_)_2_AlC *	(Nb_2/3_Y_1/3_)_2_AlC *
(Mo_2/3_Sc_1/3_)_2_AlC [35]	(Mo_2/3_Y_1/3_)_2_AlC [122]	(Mo_2/3_Zr_1/3_)_2_AlC *	(Mo_2/3_Nd_1/3_)_2_AlC [42]	(Mo_2/3_Ce_1/3_)_2_AlC [42]	(Mo_2/3_Pr_1/3_)_2_AlC [42]
(Mo_2/3_Sm_1/3_)_2_AlC [42]	(Mo_2/3_Gd_1/3_)_2_AlC [42]	(Mo_2/3_Tb_1/3_)_2_AlC [42]	(Mo_2/3_Dy_1/3_)_2_AlC [42]	(Mo_2/3_Ho_1/3_)_2_AlC [42]	(Mo_2/3_Er_1/3_)_2_AlC [42]
(Mo_2/3_Tm_1/3_)_2_AlC [42]	(Mo_2/3_Lu_1/3_)_2_AlC [42]	(W_2/3_Sc_1/3_)_2_AlC [43]	(W_2/3_Y_1/3_)_2_AlC [43]	(W_2/3_Zr_1/3_)_2_AlC *	(W_2/3_Gd_1/3_)_2_AlC [44]
(W_2/3_Tb_1/3_)_2_AlC[44]	(W_2/3_Dy_1/3_)_2_AlC [44]	(W_2/3_Ho_1/3_)_2_AlC [44]	(W_2/3_Er_1/3_)_2_AlC [44]	(W_2/3_Tm_1/3_)_2_AlC [44]	(W_2/3_Lu_1/3_)_2_AlC [44]
(W_1/3_Mo_1/3_Y_1/3_)_2_AlC [125]	(W_1/3_Mo_1/3_Gd_1/3_)_2_AlC [125]	(W_1/3_Mo_1/3_Tb_1/3_)_2_AlC [125]	(W_1/3_Mo_1/3_Dy_1/3_)_2_AlC [125]	(W_1/3_Mo_1/3_Ho_1/3_)_2_AlC [125]	(W_1/3_Mo_1/3_Er_1/3_)_2_AlC [125]
(Cr_2/3_Sc_1/3_)_2_GaC [126]	(Mn_2/3_Sc_1/3_)_2_GaC [126]	(Mo_2/3_Sc_1/3_)_2_GaC [127]	(Mo_2/3_Y_1/3_)_2_GaC [127]	(Mo_2/3_Gd_1/3_)_2_GaC [128]	(Mo_2/3_Tb_1/3_)_2_GaC [128]
(Mo_2/3_Dy_1/3_)_2_GaC [128]	(Mo_2/3_Ho_1/3_)_2_GaC [128]	(Mo_2/3_Er_1/3_)_2_GaC [128]	(Mo_2/3_Tm_1/3_)_2_GaC [128]	(Mo_2/3_Yb_1/3_)_2_GaC [128]	(Mo_2/3_Lu_1/3_)_2_GaC [128]

211 Phase: Out of plane ordering on A-site			
Nb_2_(Al_0.2_Au_0.8_)C [40]			

211 Phase: A-site solid solution			
Ti_2_(Al_0.1_Cu_0.9_)N [76]	Ti_2_(Sn_*x*_Al_1−*x*_)C(*x* = 0−1) [3]	Ti_2_(Al_1−*x*_In_*x*_)C(*x* = 0–1) [129]	Ti_2_(Sn_1−*x*_Ga_*x*_)C [130]
Ti_2_(Sn_0.9_Au_0.1_)C [41]	Ti_2_(In_0.94_Au_0.06_)C [41]	V_2_(Al_0.9_Au_0.1_)C [41]	V_2_(Ga_1−*x*_Al_*x*_)C(0.43 < *x* < 0.6) [131]
V_2_(Ga_0.91_Au_0.09_)C [41]	V_2_(Ge_0.94_Au_0.06_)C [41]	V_2_(Sn_0.67_Mn_0.33_)C [38]	V_2_(Sn_1−*x*_Fe_x_)C [38]
V_2_(Sn_0.67_Co_0.33_)C [38]	V_2_(Sn_0.67_Ni_0.33_)C [38]	V_2_(Sn_0.73_Rh_0.27_)C [41]	V_2_(Sn_0.72_Pd_0.28_)C [41]
V_2_(Sn_0.6_Ir_0.4_)C [41]	V_2_(Sn_0.6_Pt_0.4_)C [41]	V_2_(Sn_0.8_Pt_0.2_)C [41]	V_2_(Sn_0.75_Ru_0.25_)C [41]
V_2_(Sn_1−*x*_Au_*x*_)C(0 ≤ *x* ≤ 0.4) [41]	V_2_(Fe_*x*_Co_*y*_Sn_1−*x-y*_)C [38]	V_2_(Sn_0.67_Fe_*x*_Ni_*y*_Co_*z*_)C [38]	V_2_(Sn_0.67_Mn_0.167_Ni_0.167_)C [38]
V_2_(Sn_0.91_Ru_0.04_Ir_0.05_)C [41]	V_2_(Sn_0.67_Fe_0.167_Co_0.167_)C [38]	V_2_(Sn_0.67_Fe_0.167_Ni_0.167_)C [38]	V_2_(Sn_0.67_Co_0.167_Ni_0.167_)C [38]
V_2_(Sn_0.67_Fe_0.167_Mn_0.167_)C [38]	V_2_(Sn_0.67_Mn_*x*_Fe_*y*_Ni_*z*_)C [38]	V_2_(Sn_0.67_Co_0.167_Mn_0.167_)C [38]	V_2_(Fe_x_Co_y_Ni_z_Sn_1−*x*–*y*-*z*_)C [38]
V_2_(Sn_0.67_Mn_*x*_Fe_*y*_Co_*z*_)C [38]	V_2_(Sn_0.67_Mn_*x*_Ni_*y*_Co_*z*_)C [38]	V_2_(Sn_0.67_Pd_0.13_Pt_0.08_Au_0.12_)C [41]	V_2_(Sn_0.67_Mn_*x*_Fe_*y*_Co_*z*_Ni_*w*_)C [38]
V_2_(Sn_0.74_Ru_0.05_Pd_0.06_Ir_0.05_Pt_0.03_Au_0.07_)C [41]	V_2_(Sn_0.84_Ru_0.02_Pd_0.05_Pt_0.02_Au_0.07_)C [41]	V_2_(Sn_0.7_Ru_0.06_Rh_0.03_Pd_0.03_Ir_0.02_Pt_0.1_Au_0.06_)C [41]	Cr_2_(Al_0.97_Si_0.03_)C [132]
Cr_2_(Al_*x*_Ge_1−*x*_)C [133]	Cr_2_(Ga_0.4_Al_0.6_)C [131]	Zr_2_(Al_1−*x*_Sn_*x*_)C [134]	Zr_2_(Al_0.3_Sb_0.7_)C [134]
Zr_2_(Al_0.35_Pb_0.65_)C [134]	Zr_2_(Al_0.42_Bi_0.58_)C [135]	Zr_2_(Al_0.50–0.79_Bi_0.14- 0.30_Pb_0.08–0.18_)C [136]	Nb_2_(Ge_0.8_Al_0.2_)C [40]
Nb_2_(Pt_0.6_Al_0.4_)C [40]	Nb_2_(Au_0.5_Al_0.5_)C [40]	Nb_2_(Sn_0.92_Ru_0.08_)C [41]	Nb_2_(Sn_0.81_Rh_0.19_)C [41]
Nb_2_(Pd_0.5_Sn_0.5_)C [40]	Nb_2_(Sn_0.89_Ir_0.11_)C [41]	Nb_2_(Sn_0.83_Pt_0.17_)C [41]	Nb_2_(Sn_0.83_Au_0.17_)C [41]
Nb_2_(Sn_0.82_Pd_0.18_)C [41]	Nb_2_(Ag_0.3_Sb_0.4_Al_0.3_)C [40]	Nb_2_(Rh_0.2_Sn_0.4_Al_0.4_)C [40]	Mo_2_(Ga_0.33_Fe_0.5_Au_0.16_)C [137]
Hf_2_(Se_*x*_S_1−*x*_)C(*x* = 0–1) [74]			

211 Phase: X-site solid solution			
Ti_2_AlC_1−*x*_N_*x*_ [138]	Ti_2_Al(C_1−*x*_O_*x*_) (*x* < 0.5) [60]	V_2_GaC_1−*x*_N_*x*_ [78]	Nb_2_S(C_1−*x*_B_*x*_) (0 < *x* < 1) [63]
Zr_2_Se(B_1−*x*_Se_i_) (0 < *x* < 0.97) [59]			

211 Phase: Multi-site solid solution		
(Zr, Ti)_2_ (Al, Sn)C [139]	(Zr_0.8_, Nb_0.2_)_2_(Al_0.5_, Sn_0.5_)C [140]	(Ti_0.23_Zr_0.18_Hf_0.20_V_0.11_Nb_0.28_)_2_(Al_0.42_Sn_0.58_)C [141]
(Ti_0.26_Zr_0.07_Hf_0.07_V_0.47_Nb_0.13_)_2_(Al_0.66_Sn_0.34_)C [141]	(V, Nb)_2_(Sn, Fe)C [142]	(V, Nb)_2_(Sn, Co)C [142]
(V, Nb)_2_(Sn, Ni)C [142]	(V, Nb)_2_(Sn, Mn)C[142]	(Ti_2/5_V_1/5_Nb_1/3_Ta_1/5_)_2_AlC_x_N_1-x_ [143]
(Ti_1/3_V_1/6_Zr_1/6_Nb_1/6_Ta_1/6_)_2_AlC_x_N_1-x_ [143]		

312 Phase							
Ti_3_AlC_2_ [28]	Zr_3_AlC_2_ [144]	Nb_3_AlC_2_ *	Hf_3_AlC_2_ [71]	Ta_3_AlC_2_ [30]	Ti_3_SiC_2_[8]	Sc_3_SN_2_ *	Sc_3_SP_2_ *
Y_3_SN_2_ *	Zr_3_SC_2_ *	Hf_3_SC_2_ *	Ti_3_PC_2_ *	Hf_3_PC_2_ *	Ti_3_ZnC_2_ [36]	Ti_3_GaC_2_ [145]	Zr_3_GaC_2_ *
Hf_3_GaC_2_ *	Ta_3_GaC_2_ *	Ti_3_GeC_2_ [146]	Zr_3_GeC_2_ *	Hf_3_GeC_2_ *	Ti_3_AsC_2_ *	Zr_3_AsC_2_ *	Hf_3_AsC_2_ *
Sc_3_SeP_2_ *	Y_3_SeP_2_ *	Zr_3_SeC_2_ *	Hf_3_SeC_2_ *	Ti_3_PdC_2_ *	Ti_3_CdC_2_ *	Ti_3_CdN_2_ *	Zr_3_CdC_2_ *
Hf_3_CdC_2_ *	Ti_3_InC_2_ [145]	Zr_3_InC_2_ [147]	Hf_3_InC_2_ [147]	Sc_3_SnN_2_ *	Sc_3_SnP_2_ *	Ti_3_SnC2 [148]	Zr_3_SnC_2_ [148]
Hf_3_SnC_2_ [148]	Ti_3_SbC_2_ [40]	Zr_3_SbC_2_ *	Hf_3_SbC_2_ *	Sc_3_TeP_2_ *	Y_3_TeP_2_ *	Ti_3_IrC_2_ [54]	Ti_3_AuC_2_ [54]
Zr_3_AuC_2_ *	Ti_3_HgC_2_ *	Zr_3_HgC_2_ *	Hf_3_HgC_2_ *	Ti_3_TlC_2_ *	Zr_3_TlC_2_ *	Hf_3_TlC_2_ *	Sc_3_TlN_2_ *
Sc_3_TlP_2_ *	Sc_3_PbN_2_ *	Sc_3_PbP_2_ *	Ti_3_PbC_2_ *	Y_3_PbN_2_ *	Y_3_PbP_2_ *	Zr_3_PbC_2_ [149]	Hf_3_PbC_2_ [149]
Zr_3_BiC_2_ *	Sc_3_BiP_2_ *	Hf_3_BiC_2_ *					

312 Phase: M-site solid solution			
(Ti_0.5_V_0.5_)_3_AlC_2_ [106]	(Cr_1−*x*_Ti_*x*_)_3_AlC_2_ [150]	(Ti_1−*x*_Fe_*x*_)_3_AlC_2_ [45]	(Ti_1−*x*_Ni_*x*_)_3_AlC_2_ (*x* < 0.2) [45]
(Zr_1−*x*_Ti_*x*_)_3_AlC_2_ [151]	(Ti_1−*x*_Nb_*x*_)_3_AlC_2_ [152]	(Ti_1−*x*_Mo_*x*_)_3_AlC_2_ (*x* < 0.2) [45]	(Ta_1−*x*_Ti_*x*_)_3_AlC_2_ [153]
(V_1−*x*_Cr_*x*_)_3_AlC_2_ [154]	(Ti_2_V_0.75_Cr_0.25_)_3_AlC_2_ [155]	(Ti_2_V_0.9_Cr_0.1_)_3_AlC_2_ [155]	(Ti_2_V_0.5_Cr_0.5_)_3_AlC_2_ [155]
(Ti_0.67_V_0.30_Cr_0.03_)_3_AlC_2_ [155]	(TiVCrMo)_3_AlC_2_ [156]	(Ti_1−*x*_Zr_*x*_)_3_SiC_2_ *x* ≤ 0.17 [157]	(Ti_1−*x*_Zr_*x*_)_3_SiC_2_ [158]
(Ti_1−*x*_Nb_*x*_)_3_SiC_2_*x* ≤ 0.1 [159]	(Ti_0.95_Ta_0.05_)_3_SiC_2_ [160]	(Ti, W)_3_SiC_2_ [48]	(HfZrNbTiTa)_3_SiC_2_ [161]
(Ti,V)_3_GeC_2_ [117]			

312 Phase: o-MAX			
Sc_2_NbAlC_2_ *	Sc_2_TaAlC_2_ *	Sc_2_WAlC_2_ *	(V, Mn)_3_AlC_2_ [162]
(Cr_2/3_Ti_1/3_)_3_AlC_2_ [34]	(Cr_2/3_V_1/3_)_3_AlC_2_ [163]	(Mo_2_Ti)AlC_2_ [164]	(Mo_2_Sc)AlC_2_ [165]
(W_2/3_Ti_1/3_)_3_AlC_2_ *	(Cr_0.9_Mo_0.1_)_2_(Ti_0.8_Mo_0.2_)AlC_2_ [166]	(Cr_0.38_V_0.53_Ti_0.09_)_2_(Ti_0.51_V_0.49_)AlC_2_ [167]	

312 Phase: A-site solid solution			
Ti_3_(Al, Fe)C_2_ [168]	Ti_3_(Al_1−*x*_Si_*x*_)C_2_ (*x* ≤ 0.25) [169]	Zr_3(_Al_1−*x*_Si_*x*_)C_2_ [170]	Ti_3_(Al_1−*x*_Sn_*x*_)C_2_(*x* = 0.2) [171]
Ti_3_(Al_*x*_Ge_1−*x*_)C_2_ [172]	Ta_3_(Al_1−*x*_Sn_*x*_)C_2_ (*x* = 0.04) [173]	Ti_3_Si_*x*_Ge_1−*x*_C_2_ (0 < *x* < 1) [174]	Ti_3_(Si_1−*x*_Pd_*x*_)C_2_ [46]
Ti_3_(Sb_0.5_Sn_0.5_)C_2_ [40]	Ti_3_(Cd_0.5_Zn_0.5_)C_2_ [40]		

312 Phase: In-plane order on A-site			
Ti_3_(Al_1/3_Cu_2/3_)C_2_ [37]			

312 Phase: X-site solid solution					
Ti_3_Al(C_1−*x*_B_*x*_)_2_ [175]	Ti_3_Al(C_0.5_N_0.5_)_2_ [171]	Ti_3_Al(C_0.7_O_0.3_)_2_ [61]	Ti_3_GaCN [40]	Ti_3_SnCN [40]	Ti_3_SbCN [40]

312 Phase: Multi-site solid solution			
(Ti, Zr)_3_(Si,Al)C_2_ [176]	(Ti_1−*x*_Cu_*x*_)_3_(Al,Cu)C_2_ [47]	(Ti_0.65_Pd_0.35_)_3_(Si_0.9_Pd_0.1_)C_2_ [46]	Ti_3_(Sb_0.5_Sn_0.5_)CN [40]
(HfZrNbTiTa)(SiAl)_1.1_C_1.95_ [161]	(TiZrHfNbTa)_3_(AlSi)_1.1_C_1.95_ [161]	(Ti_0.23_Zr_0.31_Hf_0.31_V_0.08_Nb_0.08_)_3_(Al_0.36_Sn_0.64_) C_2_ [141]	

413 Phase							
Ti_4_AlC_3_ [177]	Ti_4_AlN_3_ [177]	V_4_AlC_3_ [178]	V_4_AlC_3-0.31_ [179]	Zr_4_AlN_3_ *	Nb_4_AlC_3_ [180]	Hf_4_AlC_3_ *	Hf_4_AlN_3_ *
Ta_4_AlC_3_ [181]	Ti_4_SiC_3_ [29]	Zr_4_PC_3_ *	Hf_4_PC_3_ *	Sc_4_SP_3_ *	Y_4_SN_3_ *	Ti_4_CuN_3_ [182]	Ti_4_ZnC_3_ *
Hf_4_ZnC_3_ *	Ta_4_ZnC_3_ *	Sc_4_GaN_3_ *	Ti_4_GaC_3_ [131]	Ti_4_GaN_3_ *	Zr_4_GaC_3_ *	Zr_4_GaN_3_ *	Nb_4_GaC_3_ *
Hf_4_GaC_3_ *	Hf_4_GaN_3_ *	Ta_4_GaC_3_ *	Sc_4_GeN_3_ *	Ti_4_GeC_3_ [183]	Ti_4_GeN_3_ *	Zr_4_GeC_3_ *	Hf_4_GeC_3_ *
Sc_4_SeP_3_ *	Y_4_SeP_3_ *	Zr_4_AsC_3_ *	Hf_4_AsC_3_ *	Ti_4_PdC_3_ *	Ti_4_PdN_3_ *	Ti_4_AgC_3_ *	Ti_4_CdC_3_ *
Ti_4_CdN_3_ *	Hf_4_CdC_3_ *	Sc_4_InN_3_ *	Ti_4_InN_3_ *	Zr_4_InN_3_ *	Nb_4_InC_3_ *	Hf_4_InN_3_ *	Ta_4_InC_3_ *
Sc_4_SnN_3_ *	Sc_4_SnP_3_ *	Zr_4_SnC_3_ *	Sc_4_SbP_3_ *	Y_4_TeP_3_ *	Ti_4_AuC_3_ *	Ti_4_AuN_3_ *	Hf_4_AuC_3_ *
Ta_4_AuC_3_ *	Ti_4_HgC_3_ *	Zr_4_HgC_3_ *	Hf_4_HgC_3_ *	Sc_4_TlN_3_ *	Sc_4_TlP_3_ *	Y_4_TlN_3_ *	Y_4_TlP_3_ *
Zr_4_TlN_3_ *	Sc_4_PbP_3_ *	Y_4_PbN_3_ *	Y_4_PbP_3_ *	Sc_4_BiP_3_ *			

413 Phase: M-site solid solution			
(Nb_0.8_Ti_0.2_)_4_AlC_3_(*x* = 0–0.3) [184]	(Ti_*x*_Ta_1−*x*_)_4_AlC_3_ [185]	(Nb_0.5_V_0.5_)_4_AlC_3_ [106]	(V_1−*x*_Mo_*x*_)_4_AlC_3_(0.325 ≤ *x* ≤ 0.675) [186]
(Nb_0.8_Zr_0.2_)_4_AlC_3_ [187]	(Ta_1−*x*_Nb_x_)_4_AlC_3_(*x* ≥ 0.25) [111]	Nb_3.9_W_0.1_AlC_3_ [188]	(Ta_1−*x*_Hf_*x*_)_4_AlC_3_(*x* ≥ 0.25) [111]
(Zr_0.75_Ti_0.25_)_4_SiC_3_ [189]	(Ti, V)_4_GeC_3_ [117]	(Ti_0.36_Nb_0.27_Ta_0.37_)_4_AlC_2.8_ [190]	(Cr_0.5_V_0.5_)_4_AlC_3_ [163]
TiVNbMoAlC_3_ [191]	TiVCrMoAlC_3_ [191]	Ti_1_V_0.7_Cr_0.05_Nb_1_Ta_1_AlC_3_ [192]	(Ti_0.28_V_0.18_Nb_0.26_Ta_0.28_)_4_AlC_2.9_ [190]

413 Phase: o-MAX						
(Cr_5/8_Ti_3/8_)_4_AlC_3_ [150]	Mo_2_Ti_2_AlC_3_ [164]	Cr_2_V_2_AlC_3_ [163]	Nb_2_Hf_2_AlC_3_ *	Mo_2_Nb_2_AlC_3_ [193]	Mo_2_Ta_2_AlC_3_ *	W_2_Ti_2_AlC_3_ *

Higher order MAX phases and other MAX phases					
Ti_5_Al_2_C_3_ [194]	Ta_6_AlC_5_ [30]	Ti_5_Si_2_C_3_ [29]	Ti_7_Si_2_C_5_ [29]	Nb_2_S_2_C [195]	Mo_2_Ga_2_C [196]
Ti_5_Ge_2_C_3_ [183]	Ti_7_Ge_2_C_5_ [183]	Ti_3_Cd_2_C_2_ [40]	Ti_7_SnC_6_ [32]	Ti_2_Au_2_C [197]	Ti_3_Au_2_C_2_ [197]
Nb_2_Bi_2_C [40]					

Solid solution of higher order MAX phases				
Ti_2.675_Nb_2.325_AlC_4_ [198]	Ti_2.5_Nb_2.5_AlC_4_ [198]	(V_0.5_Cr_0.5_)_5_Al_2_C_3_ [154]	(Mo_0.75_V_0.25_)_5_AlC_4_ [199]	Mo_4_VAlC_4_ [200]
Mo_2_(Ga_0.1_Au_0.9_)_2_C [92]	(Ti_0.22_V_0.24_Cr_0.16_Nb_0.20_Mo_0.18_)_5_AlC_4_ [201]			

^a^The symbol * means the MAXs, which are theoretically predicted to be stable but not yet synthesized by laboratory experiments

### Structure Diversity and Classification

#### Structure Classification

Herein, to more precise structural identification, MAXs are classified into three main types:

- Type I: M_*n*+1_AX_*n*_ (*n* = 1 ~ 6), hexagonal (P6_3_/mmc)
- Type II: M_*n*+2_A_2_X_*n*_ (*n* = 3, or 5), cubic (R $\overline{3 }$ m)
- Type III: M_*n*+1_A_2_X_*n*_ (*n* = 1, or 2), hexagonal (P6_3_/mmc)/ cubic (R $\overline{3 }$ m)/ hexagonal (P $\overline{3 }$ m1)

**Fig. 4:**
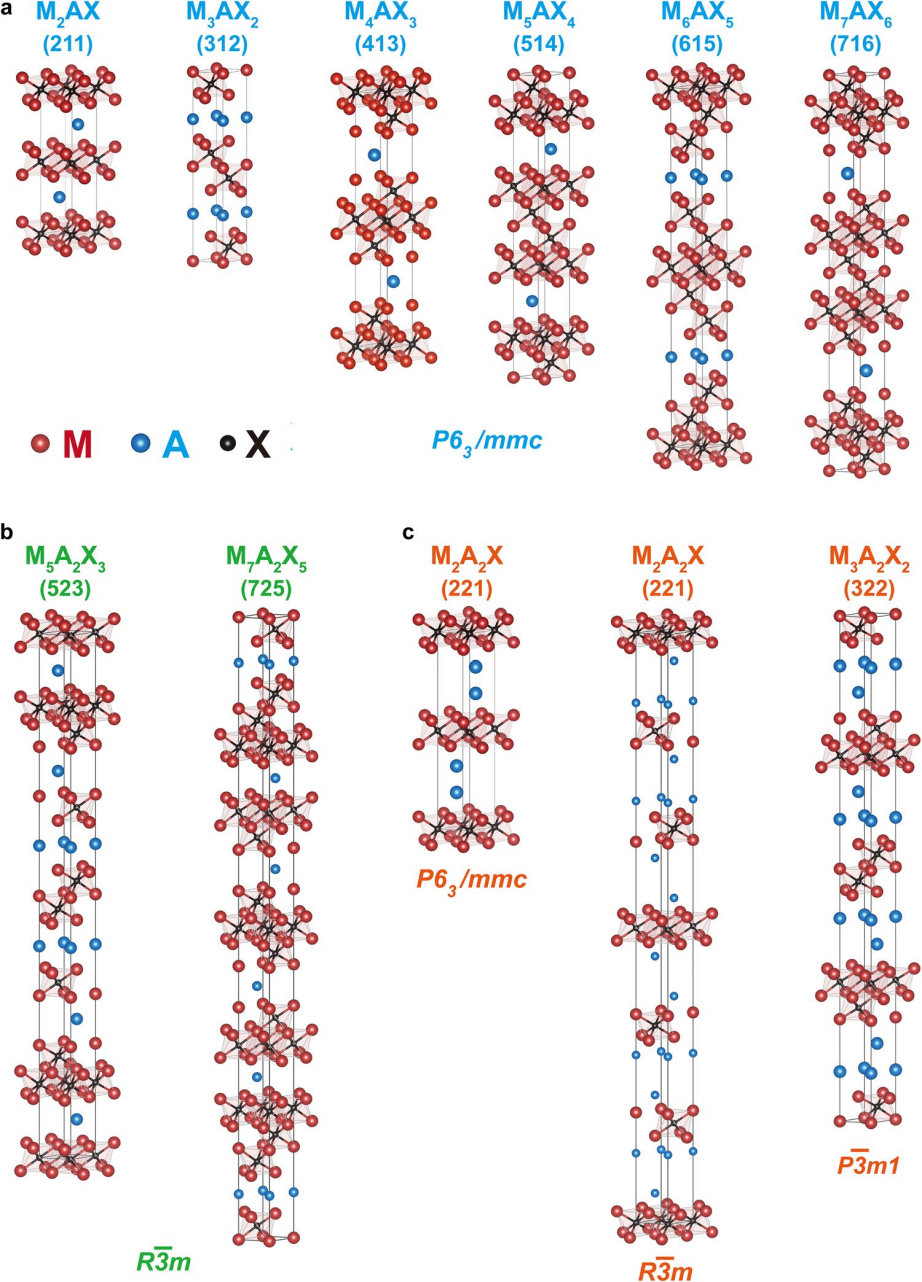
Atomic arrangement of MAX series materials. **a** Type I, **b** Type II, **c** Type III

Type I: the ternary MAXs, as M_*n*+1_AX_*n*_ (*n* = 1 ~ 6), exhibit a hexagonal layered structure within the P6_3_/mmc space group (Fig. 4a). Each X atom occupies the center of an octahedron formed by six tightly packed M atoms, with A atoms positioned between layers of M_6_X. This results in a layered structure comprising alternating M_6_X and A atom layers. *n* signifies the number of MX octahedral layers between the A atom layers; the values of *n* = 1 ~ 6 allow for further classification [1, 28, 178, 181, 202, 203]. Moreover, Mo_4_VAlC_4_ is found to be a symmetric structure of herringbone P $\overline{6 }$ m2 with the disordered solid solution [200]. The structure of (Mo_1−*x*_V_*x*_)_5_AlC_4_ was studied in depth by Snyder et al. by using high-resolution X-ray diffraction and TEM images, and the Rietveld refinement showed that the most suitable space group for (Mo_1−*x*_V_*x*_)_5_AlC_4_ is the P-6c2 rather than the conventional P6_3_/mmc space group [199].

Type II: intergrown ternary MAXs, M_5_A_2_X_3_ and M_7_A_2_X_5_, show the crystal structure's space group of R $\overline{3 }$ m due to the disrupted symmetry owing to the sequence and thickness of the alternating M_*n*+1_X_*n*_ layers. Ti_5_Si_2_C_3_ and Ti_7_Si_2_C_5_ were reported with a longer c-axis of 30.4 and 40.4 Å, respectively [29]. Type II MAXs are essentially combinations of Type I subunit cells; for instance, the 523 phase is a merger of the 312 and 211 phase subunits. The 725 phase represents a hybrid of the 312 and 413 phase subunits, with layers of 3- and 4-layer carbides alternating between A layers (Fig. 4b). To date, Ti_5_Si_2_C_3_, Ti_7_Si_2_C_5_, Ti_5_Al_2_C_3_, Ti_5_Ge_2_C_3_, and Ti_7_Ge_2_C_5_ have been identified, as Type II MAXs [29, 154, 183, 194].

Type III MAXs are defined as M_*n*+1_A_2_X_*n*_, *n* = 1 or 2. M atomic layers are spaced by double A atomic layers (Fig. 4c). A series of Mo_2_Ga_2_C, Nb_2_Bi_2_C, Ti_3_Cd_2_C_2_, Nb_2_S_2_C, Ti_2_Au_2_C, and Ti_3_Au_2_C_2_ MAX are found [40, 196, 204]. Notably, Mo_2_Ga_2_C exhibits hexagonal symmetry (space group P6_3_/mmc), akin to Type I [196, 205]. In addition, the space symmetry group of hexagonal/ P $\overline{3 }$ m1 were first identified at 1 s-Nb_2_S_2_C, and 3 s-Nb_2_S_2_C is cubic R $\overline{3 }$ m [195]. Ti_2_Au_2_C and Ti_3_Au_2_C_2_ show a trigonal crystal structure, with space group P $\overline{3 }$ m1 [197].

Objective to study structure isomerism, the M-X octahedrons are found to appear slightly deviation from their standard position. This induced a formation of *α*, *β*, and *γ* MAX polymorphs, respectively, with distinctions primarily in the stacking patterns of adjacent M-X segments [202, 206]. According to the principle of minimum energy, 211 phases exhibit a single-crystal form (*α*-M_2_AX), 312 phases exhibit two (*α*-M_3_AX_2_ and *β*-M_3_AX_2_), and 413 phases exhibit three (*α*-M_4_AX_3_, *β*-M_4_AX_3_, and *γ*-M_4_AX_3_). A-layer atomic slippage induces structural transformation from *α* to *β* to *γ* MAXs, accompanied by changes in atomic positions. For detailed atomic occupancy information, please refer to Chapter 2 in “MAX Phases: Properties of Machinable Ternary Carbides and Nitrides,” Michel W. Barsoum [207].

#### Solid Solutions

**Fig. 5:**
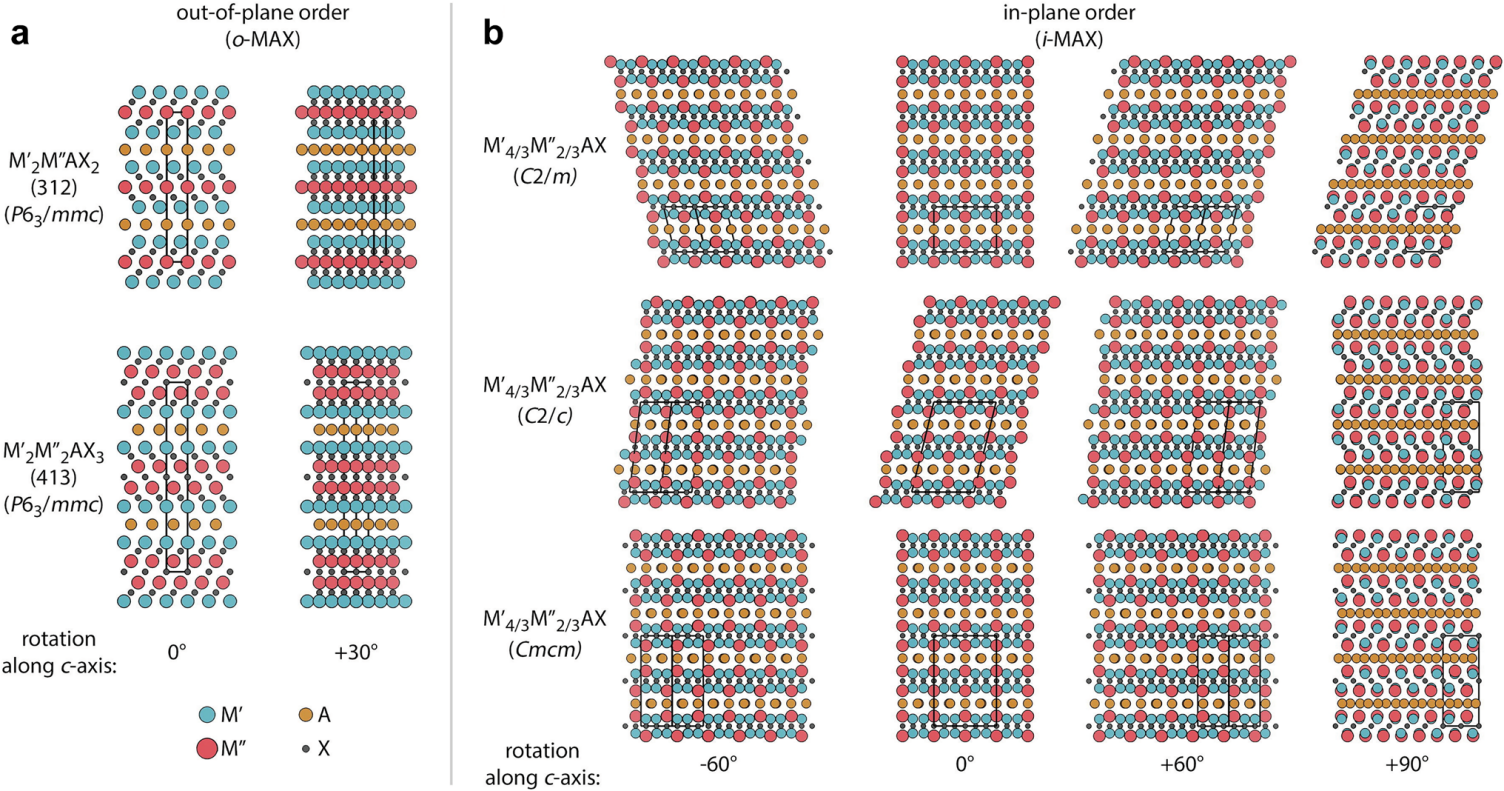
Ordered MAX series materials. **a** Out-of-plane ordered 312 and 413 o-MAX; **b** in-plane ordered i-MAX. Reproduced with permission from Ref.[208]. Copyright 2023, Elsevier

Multi-element occupations at the M, A or X sites create the solid solution MAXs in Table 1. Due to the mutual modulation between various elements, these atoms show two kinds of arrangement states: disordered and ordered. An ordered arrangement is that each M′ and M′′ atom occupies, respectively, a separate atomic layer and shows the out-of-plane ordered structure. Within a single atomic layer, there is only one type of M atom. M′ atomic layers envelop one or two layers of M′′ atomic layers (as shown in Fig. 5a). This type of ordered solution of MAXs is marked as o-MAXs and remains hexagonal (P6_3_/mmc) [164]. The ideal o-MAXs of 312 M'_2_ M''AX_2_ and 413 M'_2_ M''_2_AX_3_ present a relatively accurate proportion of M'/M'' [208]. Recently, the third metal element was introduced as doping atoms in M'/M''-site in disordered form [166]. A series of correlations were discovered, (i) M' near the A atomic layer does not form the corresponding binary rock salt MC structure, (ii) M' and M'' atomic sizes are similar, and (iii) the electronegativity between M' and A is different [209, 210]. The other ordered arrangement is that M′ and M′′ atoms appear in the same atomic layer and exhibit the in-plane ordered structure (i-MAX) [35]. It is worth noting that i-MAX showcases a blend of monoclinic (C2/m and C2/c) and orthorhombic (Cmcm) crystal structures (as shown in Fig. 5b). The deviation from hexagonal symmetry in the i-MAX structure arises from the atomic size difference between the two metals being greater than 0.2 Å (rM' < rM''), which causes the M' and M'' atoms to no longer occupy the same planes. The M'' atoms move closer to the A-layer, influencing the structure of the A-layer and causing it to deviate from a hexagonal lattice toward a Kagomé-like lattice. However, the three types of i-MAX structures still maintain the same Al-M'_4/3_ M''_2/3_-Al subunit, with only the stacking arrangement along the c-axis differing [122]. The solid solution orderly structure of i-MAXs is significantly influenced by the mass and atomic radii of their constituent elements. The i-MAX enriched with lighter elements like Ce and Pr tends to adopt the C2/m structure, while those with heavier elements such as Tb, Nd, Gd, Dy, Ho, Er, and Tm favor the C2/c structure [42]. It is driven by atomic radius differences, particularly among M elements with larger radii. The varying distances of M elements to A elements and the structural configurations of M′ and M′′ elements are key factors in this differentiation [43, 52].

Explorations into A-site and X-site solid solutions offer a strategic avenue for tuning the structures and functionalities of MAXs. Ge, Fe, Co, Ni, Mn, Au, Pt, Ru, Sb, Ir, Pd, Rh, Bi, and Cu are introduced into A-site [38, 40, 41]. Dual-site solid solution MAXs, where element solid solutions occur at two sites among M, A, or X, with those sites occupied by multiple elements, predominantly take place at the M- and A-sites. This is because the X site is usually filled by C and N [78, 138], while a broader selection of elements for M- and A-sites facilitates the formation of dual-site solutions [141, 143].

There is no doubt that great achievements have been made in the study of the element and structural diversity of MAXs, which laid the foundation for the development of properties and functions. At the same time, it also made us realize that MAXs is an extremely complex material system, and it is necessary to systematically understand the internal relationship between its elements and structures.

## Synthesis Strategy

MAXs’ synthesis is a multi-level and complex process, involving multiple physical and chemical phenomena such as atomic diffusion, chemical bond breaking and formation, and so on, which lies in the reconstitution of chemical bonds and atomic structures to a specific layered structure. M-X bonds help maintain the structure's stability, while the weak M–A bonds provide a large degree of freedom for the diffusion of A atoms. This weak bond property enables rapid migration of A atoms, which promotes the formation of the MAXs. The synthesis strategies of solid, molten salt, and vapor systems are described.

### Solid-State Reaction

**Fig. 6:**
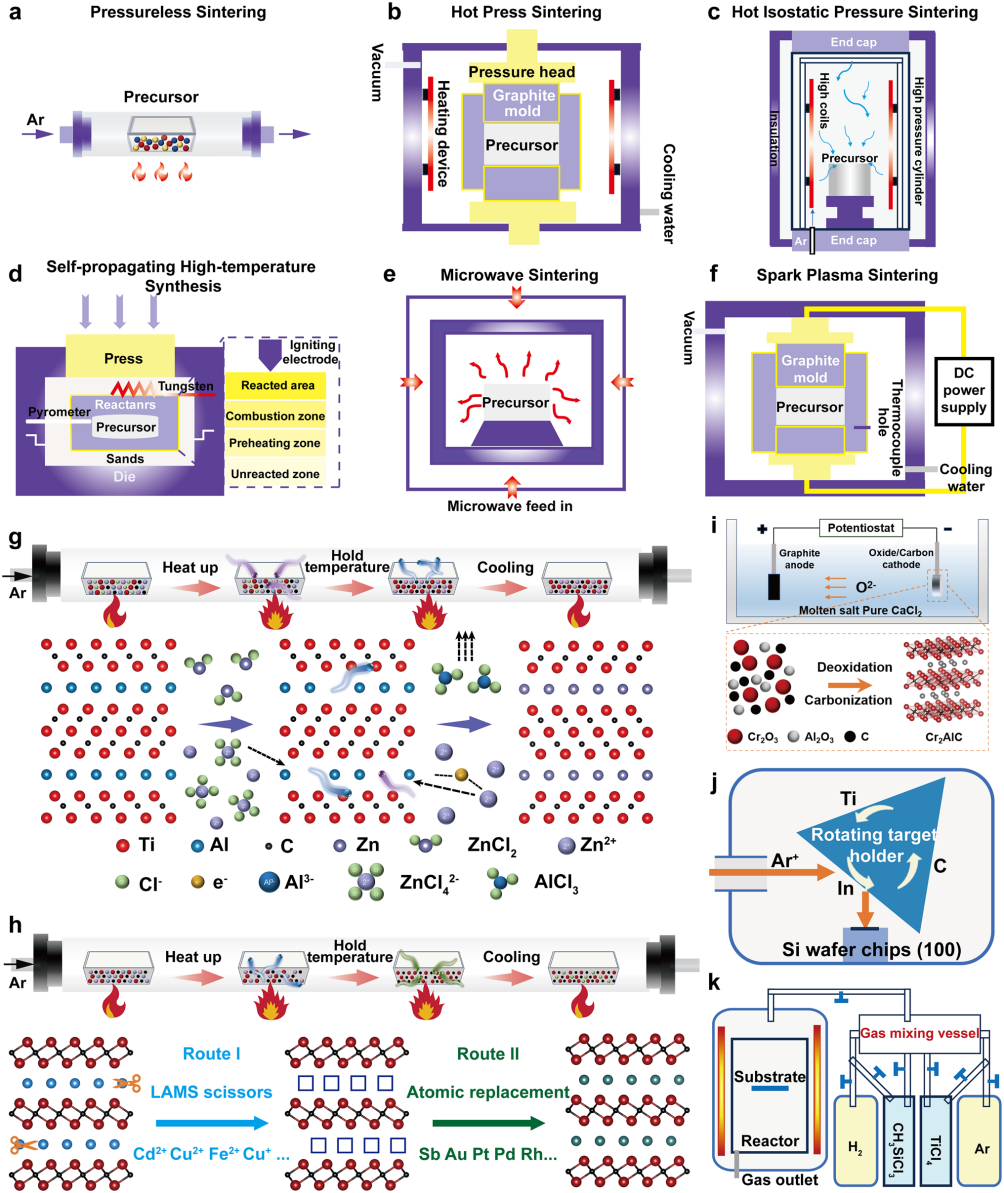
**a** Pressureless sintering. **b** Hot press sintering. **c** Hot isostatic pressure sintering. **d** Self-spreading high-temperature synthesis. **e** Microwave. **f** Spark plasma sintering. **g** Lewis acidic molten salt routes. **h** Structural editing based on chemical scissor-mediated intercalation protocol. **i** Molten salt electrolysis. **j** Ion beam sputtering. **k** Low-pressure CVD system

Solid-state reaction sintering typically employs solid powder particles including M powders or their metal hydrides, elemental A metal powders, graphite powders, and metal nitrides, as the precursors. Relying on a high-temperature and pressure environment (Fig. 6a–f), the diffusion kinetics of the constituent atoms is accelerated, and form M_*x*_X_*y*_, or M_*x*_A_*y*_ at the interface of these precursor particles. Upon increased temperature, M_*x*_X_*y*_ and M_*x*_A_*y*_ react to form MAX.

#### Pressureless Sintering

Pressureless sintering employs high-temperature devices like tubes and muffle furnaces (Fig. 6a). The precursor's particle size, chemical stoichiometry, heating rate, peak temperature, and duration are critical for MAXs formation. This method produces MAXs with lower densities, facilitating their conversion into powders. Its benefits include straightforward operation, versatile precursor selection, and adaptability for mass production. However, it requires extended duration at high temperatures, results in lower densities, and necessitates ball milling for particle size adjustment. This approach has been successfully applied in the synthesis of materials such as V_2_SnC, Ti_3_AlC_2_, Ti_3_GaC_2_, among others [28, 89, 145, 211]. Carbon materials can be employed to reduce metal oxides at high temperatures. Therefore, these conventional oxides are expanded as the precursor powders of MAXs. Utilizing Cr_2_O_3_, V_2_O_5_, Ga, Ge, and C, a series of high-purity MAXs (Cr_2_GeC, Cr_2_GaC, V_2_GeC) are prepared; the initial carbon content crucially influenced Cr_2_GaC's conversion rate [212]. Ti_3_SiC_2_ is also prepared by TiO_2_ and SiO_2_, highlighting cost-effectively [213].

#### Hot Press Sintering

Hot pressure is introduced via a hot press furnace. The process involves ball milling precursors for uniform mixing, followed by hot pressing (low pressure at 1000–1500 °C and high pressure at 1200–2000 °C) to aid the synthesis and densification processes (Fig. 6b). This technique's merit lies in its ability to directionally advance precursor reactions under pressure, preventing precursor loss through sublimation in a sealed environment, making it ideal for creating dense MAX bulk materials. However, this technology also has some drawbacks, such as potential carbon pollution from graphite molds, scalability challenges, and high stability requirements for equipment due to long-term high-pressure conditions [70].

#### Hot Isostatic Pressure Sintering

Hot isostatic pressing sintering uses an inert gas as a pressurizing medium in a high-pressure environment. The workflow involves ball milling to blend precursor powders, pre-pressing into solid bulks, sealing in inert gas, and then sintering. Operating within a temperature range of 1000–2000 °C and under inert gas pressures reaching 200 MPa, this method ensures uniform compression of MAX at high temperatures and pressures, resulting in superior density and uniformity (Fig. 6c). This technique is valued for its rapid production time, streamlined process, reduced energy usage, and lower material wastage. However, the reaction scalability of this method is limited due to the requirement of encapsulating the precursor powder in a specific glass or metal container [88].

#### Self-Spreading High-Temperature Synthesis

Relying on the exothermic reaction, self-spreading high-temperature synthesis leverages to facilitate solid-state reactions. The procedure involves pre-pressing precursor materials into compact particles, igniting these particles with tungsten or molybdenum wire in a vacuum to avoid oxidation, and conducting the self-propagating sintering process where temperatures can soar up to 2000 °C, with combustion wave speeds reaching 25 cm s^−1^. This leads to the creation of porous MAX particles **(**Fig. 6d) [214, 215]. The benefits include its straightforward execution, fast reaction, and minimal energy requirements. Nonetheless, it faces challenges such as difficulty in controlling the reaction, a high and uncontrollable amount of secondary phases, and poor repeatability.

#### Microwave

Microwave heating’s rapid process stems from the intense interaction between solids and microwave radiation, reaching exceedingly high temperatures (Fig. 6e). Despite its advantages of easy operation, fast reaction speed, and high cost-effectiveness, microwave sintering still faces many bottlenecks that need to be overcome, such as the type limitations of MAX, difficulties in thermal management, precision issues in temperature monitoring and control, uneven heating, cracking of sintered parts, and challenges of uniform heating over large areas [105, 109, 203].

#### Spark Plasma Sintering

Spark plasma sintering employs electric currents and localized high-temperature heating to foster plastic deformation and diffusion among precursor powders, facilitating bonding and sintering (Fig. 6f), which were utilized for the preparation of Zr_3_InC_2_, Hf_3_InC_2_, Zr_3_SnC_2_, and Hf_3_SnC_2_. Spark plasma sintering combines plasma activation, hot pressing, and resistance heating to offer benefits such as quick temperature escalation, brief sintering durations, lower temperatures, and grain uniformity, aiding in precise microstructure control and achieving high-density materials. Despite its operational simplicity and repeatability, its drawbacks include significant energy demands, complex machinery, challenging maintenance, and elevated equipment costs [147, 148, 216].

### Melting Reaction

#### Molten Salt Sintering

The molten salt sintering technique leverages the flow properties of low melting point salts to enhance the delivery and spread of precursor materials for MAXs, improving the interaction among reactants to control reaction kinetics, the nucleation and growth processes [87]. These key procedures include: (1) the types of molten salts; (2) sintering temperature, rate, and duration; (3) isolation and purification of products. The selection of an appropriate molten salt is pivotal; the salt's melting point should be lower than the metal precursors to ensure a liquid state; in addition, the cost-effectiveness, solubility in water, and the diffusion rate of reactants are considerable. Furthermore, an inert environment can prevent oxidation of metal precursors. The advantage lies in high purity, uniform size, and low sintering temperature based on recycled molten salt. However, there are disadvantages such as high cost and environmental pollution. Future research will focus on environmentally friendly molten salts, sintering optimization, and functional ceramic development [217, 218]. In addition, molten salts are also employed as electrolytes to assist the electrochemical synthesis of MAXs (Fig. 6i) [219].

#### Lewis Salt Substitution Strategy

Lewis acid molten salts (LAMS) enable the A-site atoms to bond with the molten salt's anions, while the molten salt's cations migrate into the vacancies left by the A atoms (as shown in Fig. 6g). To obtain high-quality MAXs, these processes should be strictly controlled: (1) the proportion of MAX and LAMS; (2) the reaction temperature and environment; (3) the separation and purification of products. Based on the LAMS, a series of MAXs with new A-sites are prepared, such as Ti_3_ZnC_2_, Ti_2_ZnC, Ti_2_ZnN, and V_2_ZnC [36], Ti_2_(Al_*x*_Cu_1−*x*_)N and Nb_2_CuC, Ti_4_CuN_3_ [182]; some transition metals, like Fe, Co, Ni, Cu, etc. are incorporated into new MAXs via homologous substitution reactions [40]. Meanwhile, an innovative method of interlayer chemical reaction mediated by "chemical scissors" was further reported, significantly expanding the element types of MAXs, as shown in Fig. 6h. Route I: LAMS cations act as "chemical scissors" to etch A-site atoms of MAXs, opening non-van der Waals gaps and forming interlayer atomic vacancy structures; Route II: solvated intercalation atoms in molten salt diffuse into interlayer atomic vacancies to form MAXs. The synergistic effect of the "chemical scissors" and the guest ions offers greater space for interlayer composition and structural regulation, resulting in a series of new MAXs containing conventional A-site elements (Al, Ga, In, and Sn) and unconventional A-site elements (Bi, Sb, Fe, Co, Ni, Cu, Zn, Pt, Au, Pd, Ag, Cd, and Rh) [220]. Lewis salt replacement strategy realizes the structure editing of MAXs, interlayers unconventional elements into the A atomic layer of MAXs, breaks through the traditional metallurgical reaction bound, and expands the types and application range of MAX family. However, due to the limited types of Lewis salt, the complex reaction process and high cost make large-scale preparation impossible. In the future, how to develop the new Lewis salts, further study the reaction mechanism, and optimize the sintering process is crucial to form new quality productivity based on MAXs.

### Vapor Deposition

#### Physical Vapor Deposition

Physical vapor deposition (PVD) is reported for preparing MAX thin films with high purity, controllable composition, and wide applicability. Under high vacuum conditions, PVD can effectively avoid the introduction of impurities and achieve precise control of the thickness and composition of films. As shown in Fig. 6j, the processes include (1) the selection of substrates and MAX targets; (2) PVD deposition of the thin film under a protective atmosphere; and (3) annealing treatment. However, due to the specific crystal structure required for MAXs, PVD-deposited films often exhibit amorphous or mixed phases and require high-temperature annealing (usually 600–1200 °C) to crystallize [221, 222]. In addition, the high equipment requirements limit the large-scale production of PVD, and the internal stress during the deposition process affects the quality and adhesion of the films.

#### Chemical Vapor Deposition

Chemical vapor deposition primarily involves creating thin films by chemical reactions of gaseous compounds or elements on the substrate surface. The process entails several critical steps: (1) selecting and cleaning the substrate is pristine to ensure a clean surface; (2) choosing the appropriate reactive gases to match the MAX targets’ requirements; (3) managing the reaction by placing the substrate in a reaction chamber, introducing selected gases, and heating to the desired temperature; (4) modifying deposition rates and film quality by adjusting the deposition duration and gas flow; (5) cooling the films. A mixture of TiCl_4_, SiCl_4_, CCl_4_, and H_2_ gases are employed to fabricate polycrystalline Ti_3_SiC_2_ MAX films [223] (Fig. 6k).

383 variants with diverse elemental compositions, and crystalline structures are prepared through methods like reaction sintering and molten salt techniques. These methods can precisely manipulate the microstructure, shape, and defects of MAXs. Efforts are ongoing to enhance the purity of the outcomes, boost preparation efficiency, streamline the process, and cut down on energy use and environmental impact.

Despite the growing variety of methods to prepare MAXs, the process encounters several hurdles. Primarily, the synthesis of MAXs requires high-temperature and high-pressure environments, posing a challenge for scaling up and industrial production. Synthesis often occurs at temperatures ranging from 1000 to 1700 °C and pressures from 1 to 50 MPa, necessitating special equipment and techniques that increase costs and risks. Frequently, the synthesis results in incomplete reactions, leading to products with impurities and defects that compromise their purity and functional properties. For instance, excessive reactions between the M element with A or X elements can result in unwanted MA or MX phases, or internal diffusion of the A element can disrupt the A-layer structure, diminishing the electrical and thermal conductivity and the oxidation resistance of MAXs. Moreover, controlling MAXs' geometrical morphology and crystal structure is challenging, limiting their utility. Typically as powders or bulks, it is challenging to fabricate MAXs into coatings, films, or fibers. Their layered structure complicates the creation of heterogeneous or composite configurations, thus limiting their potential applications across various application scenarios.

## Simulation and Prediction

Due to their intricate crystal structures and complex elemental makeup, high costs, low efficiency, limitations on shape, harsh synthesis conditions, and complex equipment hindered the advancement of novel MAXs. Simulation and prediction can aid scientists in delving into the physical and chemical essences and linking composition, structure, and properties. The synergy between experimental validation and computer simulation enriches the developmental insights and guidance for MAXs. Expedited exploration of new MAXs necessitates the leverage of supercomputing power. Techniques such as introducing new elements, cluster expansion, random crystal structure prediction, and evolutionary algorithms open up new avenues in understanding MAX structures, compositions, and properties. It is crucial to explore the MAXs with unknown element compositions and new structures based on the thermodynamic stability principle.

### Prediction Types of MAXs

Currently, high-precision computational methods are extensively employed, including density functional theory for electronic structure calculations, Monte Carlo simulations, molecular dynamics simulations, phase field methods, and finite element analysis. With the continuous development of MAXs experimental research and theoretical foundations, a large amount of observation and simulation data has been obtained through these methods. By utilizing these extensive datasets, machine learning techniques have provided more accurate and efficient predictions for the new MAXs. The approach promises to greatly speed up the design process of new materials and shorten the time needed for materials to be converted from laboratory research to industrial applications. Through their training and optimization, machine learning models offer enhanced understanding and forecasting of MAXs' performances and behaviors, marking a novel and efficient avenue for advancing materials science research and development.

**Fig. 7:**
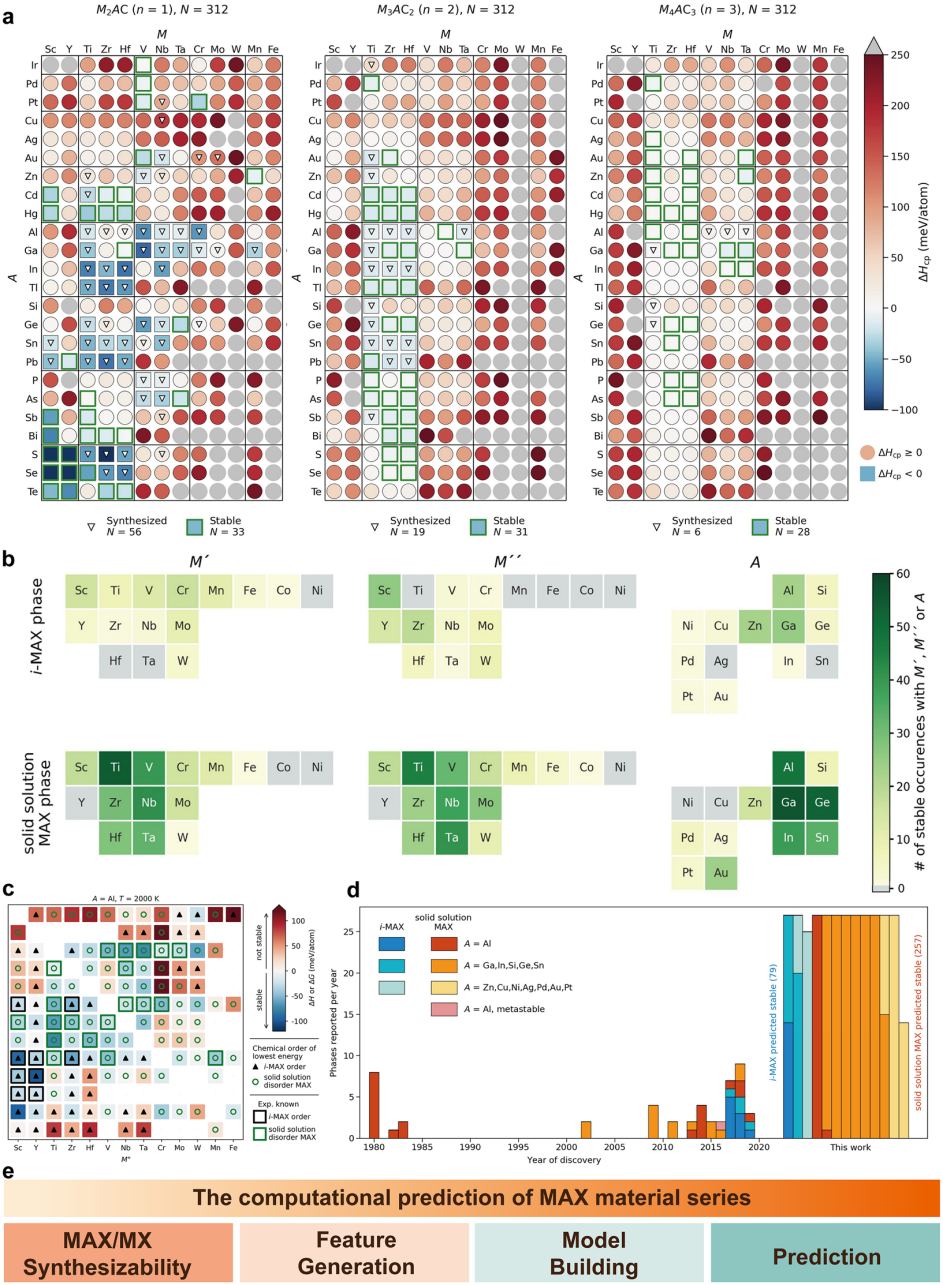
**a** Calculated stability for C-based MAX. Reproduced with permission from Ref. [208]. Copyright 2023, Elsevier. **b** Element distribution maps in predicted stable i-MAX and solid solution MAX series materials. Reproduced with permission from Ref. [229]. Copyright 2022, Royal Society of Chemistry. **c** Predicted phase stability for (M′_2/3_ M′′_1/3_)_2_AlC alloys. Reproduced with permission from Ref. [229]. Copyright 2022, Royal Society of Chemistry. **d** Statistical chart of stable MAX series materials formed from experimental implementation and theoretical prediction since 1960. Reproduced with permission from Ref. [229]. Copyright 2022, Royal Society of Chemistry. **e** MAXs calculation workflow diagram

This research methodically examined the MAXs' structural stability, lattice parameters, mechanical characteristics, electronic properties, and thermal conductivity using density functional theory principles. These analyses provide a theoretical basis for identifying promising MAXs and have informed experimental synthesis efforts [209, 224–227]. In 2021, Khaldi Alidusti et al. [228] utilized density functional theory to analyze 1122 MAX candidates and found that 466 MAX and 26 MXene may be prepared. In 2023, Martin et al. [208] conducted a more detailed investigation into the phase stability of MAXs. Figure 7a shows the stability heat maps of the C-based MAX. The 3705 different MAXs, with various combinations of M, A, and N, B, and P elements, were evaluated based on the stability and identified 180 ternary MAXs that were theoretically predicted to be stable but not synthesized in the laboratory. In 2022, Dahlqvist et al. [229] utilized DFT and PBE-parameterized GGA for insights into electronic exchange and correlation within MAXs (Fig. 7b). Notably, there are 23 thermodynamically stable i-MAX, with 9 already verified experimentally, and highlighted 48 stable disordered solid solution MAXs (Fig. 7c). The synthesis and theoretical predictions of MAXs are counted (Fig. 7d), illustrating how the ordered or disordered nature is influenced by the size disparity between M- and A-site elements [210].

### Functional Development of MAXs

Benefiting from ceramic and metal features and their characteristics like low density, high modulus, excellent electrical and thermal conductivity, thermal shock resistance, and resistance to high-temperature oxidation, MAXs demonstrate exceptional potential for applications under extreme conditions such as high temperatures, severe corrosion, and radiation exposure. The diversity of MAXs, however, introduces significant challenges in researching their properties, with the current lack of comprehensive and systematic studies hindering broader application. Through in-depth knowledge of factors like composition, microstructure, crystal structure, and processing parameters, combined with elements' physical and chemical properties, leveraging theoretical material science to create physical models and mathematical calculations enables effective prediction of MAXs' performance parameters (Fig. 7e).

In 2016, Wang et al. [230] employed density functional theory-based first-principles calculations to thoroughly investigate the lattice structure, stability, electronic structure, and mechanical and thermal properties of Ti_3_(Sn_*x*_Al_1−*x*_)C_2_ solid solutions across varying Sn concentrations. Their research indicates that increasing Sn content minimally impacts the crystal structure, and these solid solutions behave as metallic, stable, and brittle materials both thermodynamically and mechanically. Notably, the maximum bulk modulus was observed at the Sn doping concentration of 0.75, and the maximum shear modulus was observed at the Sn doping concentration of 0.5. Moreover, these solid solutions boast high melting points and Debye temperatures, with their lattice thermal conductivity at room temperature exceeding 40 W m^−1^ K^−1^ for x values of 0, 0.25, and 0.5, indicating superior thermal conductivity. In 2021, Ahams et al. [231] first applied DFT to analyze the structure, elasticity, and electronic properties of novel MAXs such as (V_0.25_Zr_0.75_)_2_PbC, (V_0.5_Zr_0.5_)_2_PbC, (V_0.75_Zr_0.25_)_2_PbC, and V_2_PbC and studied the effects of changes in V and Zr concentrations on the properties of Zr_2_PbC. Their research revealed that the structural integrity of these new MAXs remains stable within the P6_3_/mmc space group as the V element ratio increases, with the 25% V-containing samples showing improved plasticity, compressibility, brittleness, and hardness. Elastic constants rose with higher V concentrations, and the atomic concentration also influenced the MAXs' electronic band structure and total density of states (TDOS), offering crucial insights for predicting and understanding the performance of MAXs. In 2022, Zeng et al. [232] employed density functional theory (DFT) to explore the Nb_2_AN (*A* = Si, Ge, Sn) MAXs compounds, focusing on their structure, mechanical attributes, electronic structure, and thermal behavior. The study revealed that these compounds not only maintain strong structures but also maintain dynamic mechanical stability. Notably, the Nb_2_SnN phase stood out for its superior thermal shock resistance, even though it didn't have the highest melting point among the group. Due to its thermal expansion coefficient in the temperature range of 300–1452 K being very close to that of nickel-based alloys, coupled with the lowest lattice thermal conductivity, it has become a promising candidate for thermal barrier coating (TBC) applications. The Nb_2_SnN phase is distinguished by its mechanical resilience, attributed to the minimal deformation of its octahedral structure, high ductility, and low anisotropy. Electronic analyses pinpointed the phase's low Debye temperature Θ to its high ionic character and minimal covalency. Further extending the scope, in 2024, Tian et al. [233] delved into the impact of pressure on V_2_ZnC's crystal structure, elasticity, electronic framework, and thermodynamic steadiness through DFT investigations. They discovered that V_2_ZnC transitions from brittleness to ductility at a pressure of 20 GPa, with its elastic constants and modulus escalating in response to increased pressure.

These findings underscore the pivotal role of theoretical computations in paving the way for novel materials, enabling the anticipation of diverse material characteristics such as optical, magnetic, and electronic transport properties. Through advanced simulations, scientists gain deeper insights into materials' band structures, Fermi levels, and electron density distributions, which facilitate predictions about their performance under specific conditions. These insights are invaluable to material developers, which guide the selection of material composition, synthesis methods, and processing parameters, thereby simplifying the creation and optimization of new materials.

## Properties and Performances

MAXs demonstrate mechanical properties, thermal properties, electrical properties, magnetism, high-temperature oxidation resistance, and corrosion resistance, owing to their layered structure consisting of alternating M-X layers bonded by strong covalent bonds and M-A layers bonded by weak metal bonds, endowing them with high hardness, strength, toughness, and excellent electrical and thermal conductivity. This structure enables the material to maintain good mechanical and chemical stability even at high temperatures. For specific application fields, the performance of MAXs can be further adjusted and optimized through methods such as alloying, nanomaterialization, and surface modification.

### Mechanical Properties

MAXs exhibit a unique combination of mechanical advantages, including high strength, moderate hardness (4–6 GPa), excellent fracture toughness (3–5 MPa m^1/2^), superior wear resistance, and exceptional thermal shock resistance. These performances can be maintained even at high temperatures due to the stable layered crystal structure. The mechanical properties stem from the hybrid bonding, with strong covalent M-X bonds contributing to hardness and high-temperature stability, and metallic M-A bonds providing ductility. The layered structure also allows for self-lubrication and crack resistance, ensuring enhanced durability. This unique interplay of ceramic-like and metallic features gives MAXs a significant edge in demanding applications like aerospace, automotive, and energy systems. Table 2 summarizes the mechanical properties.

**Table 2:** Mechanical properties of MAXs at RT

MAX Phases	Density [g cm^−3^]	Vickers hardness [GPa]	Young’s modulus [GPa]	Flexural strength [MPa]	Compressive strength [MPa]	Fracture toughness [MPa m^1/2^]
*211 phase*						
Ti_2_SC [234]	4.6	*	290	*	*	*
Ti_2_AlC [235]	4.1	5.8 $\pm$ 0.5	277	432 $\pm$ 12	952 $\pm$ 6	6.5 $\pm$ 0.2
Ti_2_SnC [236]	4.7	3.5 $\pm$ 0.4	*	*	*	*
Ti_2_AlN [237]	4.3	*	285	*	*	*
Ti_2_AlC_0.5_N_0.5_ [237]	4.2	*	290	*	*	*
V_2_AlC [66]	4.0	2.2 $\pm$ 0.1	235	270 $\pm$ 12	527 $\pm$ 12	5.7 $\pm$ 0.2
Cr_2_AlC [67, 238]	5.17	4.9	282	469 $\pm$ 27	949 $\pm$ 22	6.2 $\pm$ 0.3
Cr_2_GeC [239]	5.2	*	208	*	*	*
Nb_2_AlC [69]	6.44	4.5 $\pm$ 0.3	294	481 $\pm$ 42	*	5.9 $\pm$ 0.3
Nb_2_SnC [236]	8.0	3.8 $\pm$ 0.2	216	*	*	*
Ta_2_AlC [70]	11.46	4.4 $\pm$ 0.1	292	360 $\pm$ 19	804	7.7 $\pm$ 0.2
Zr_2_SnC [236]	6.9	3.9 $\pm$ 0.3	178	*	*	*
Hf_2_SnC [236]	11.2	3.5 $\pm$ 0.4	237	*	*	*
*312 phase*						
Ti_3_SiC_2_ [8]	4.5	4.0	320	260 $\pm$ 20	600	*
Ti_3_AlC_2_ [235]	4.21	2.7–3.2	297	340	760	6.9–7.2
Ti_3_GeC_2_ [240, 241]	5.22	5.0	340	*	1277	*
Ti_3_(Si,Ge)C_2_ [241]	5.02	*	322	*	*	*
Ti_3_AlCN [237]	4.5	*	330	*	*	*
*413 phase*						
Nb_4_AlC_3_ [242]	6.97	2.6 $\pm$ 0.2	306	346 $\pm$ 38	515 $\pm$ 44	7.1 $\pm$ 0.3
Ta_4_AlC_3_ [243]	13.18	5.1 $\pm$ 0.1	324	372 $\pm$ 20	821 $\pm$ 97	7.7 $\pm$ 0.5
Ti_4_AlN_3_ [243]	4.6	2.5	310 $\pm$ 2	350 $\pm$ 15	475 $\pm$ 15	*

^a^The symbol * indicates that MAXs data have not yet been reported

Typically, MAXs have a brittle-plastic transition temperature (BPTT), which is the transition temperature from typical brittle fracture (traditional ceramics) to fracture toughness (metals). When the environment temperature is higher than BPTT, the bending strength rapidly decreases. As the temperature increases, the Young's modulus of MAXs decreases, but the high stiffness remains [66]. Thermal stability is also an important criterion. MAXs can sustain the structure integrity, and the strength increases upon quenching in the air at 1300 °C. In addition, larger grain sizes can achieve higher thermal stability [244]. Attributed to the microplastic behavior and quasi-metallic damage tolerance (KBs) during quenching, MAXs can maintain excellent mechanical properties and thermal stability even in high-temperature environments.

### Thermal Properties

#### Thermal Conductivity

MAXs are good thermal conductors, with thermal conductivities ranging from 12 to 60 W m^−1^ K^−1^ at RT. The total thermal conductivity (κ_th_) is determined by both the electronic thermal conductivity (κ_e_) and the phonon thermal conductivity (κ_ph_). In general, for non-S- or Al-containing MAXs, the phonon thermal conductivity (κ_ph_) is lower than the electronic thermal conductivity (κ_e_). However, MAX containing S and Al is good phonon conductors; the κ_ph_ value of Ti_3_AlCN at RT is up to 36 W m^−1^ K^−1^, the highest value in MAXs [245]. MAX ' κ_ph_ is related to their defect concentration, which can be evaluated by the residual resistance ratio (RRR). As the RRR value increases, the κ_ph_ value increases [246]. However, the point defects and the rattler effect suppress the contribution of κ_ph_ to the thermal conductivity in part of MAXs, which refers to the vibration atoms at their equilibrium positions leading to phonon scattering. Many elements, with atomic numbers > S, tend to "rattle," which explains why the phonon thermal conductivity of Ti_2_InC, Hf_2_InC, Nb_2_SnC, and other compounds contributes less to the overall thermal conductivity.

#### Heat Capacity and Thermal Expansion Coefficient

The heat capacity of MAXs depends on the following factors: temperature, chemical composition, crystal structure, and potential phase transitions. At low temperatures, the heat capacity increases nonlinearly, governed by the Debye model, while at high temperatures it approaches the classical limit (~ 3R per atom). At high temperatures, it tends to be constant, approaching the Dulong Petit limit. Variations in M, A, and X significantly influence phonon spectra and thus heat capacity. The layered structure results in unique lattice vibrations, with defects and doping further modifying thermal properties. Despite metallic behavior, MAXs exhibit low electronic contributions to heat capacity, with phonons being dominant. These properties, combined with high thermal conductivity and stability, make MAXs suitable for high-temperature applications such as thermal management, energy storage, aerospace, and nuclear systems [7, 8].

The coefficient of thermal expansion (CTE) describes the variation in volume with temperature. A low CTE can reduce internal stress caused by thermal expansion and contraction, thereby improving the thermal cycling stability and service life. The thermal expansion behavior is anisotropic due to the relatively weak interlayer bonds (MA or van der Waals forces) and relatively strong intra-layer bonds (MX). This unique bonding characteristic limits the thermal expansion of the lattice, allowing MAX to maintain stable volume in high-temperature environments and reduce the damage of thermal stress to the structure [8].

### Electrical Properties

#### Resistivity

MAXs exhibit metallic conductivity because: (1) The high density of electronic states near the Fermi level provides a large number of conductive electrons. (2) The unique layered structure of MAX, alternating M-X layers and A layers, facilitates the free electron migration within the M-X layers, while reducing scattering and thereby enhancing conductivity. (3) The weak interlayer interactions result in lower electron scattering rates, thereby maintaining higher electron mobility. Meanwhile, the scattering effects of impurities, vacancies, or other defects may lead to a higher residual resistivity and a lower RRR at low temperatures. The solid solution MAXs show a higher resistivity than the corresponding MAXs due to the stronger scattering effect, leading to a decrease in electron mobility. Since N(EF) predominates in the d-orbitals of the solid solution elements, the impact of substitutions at different positions (M, A, X) on resistivity is not equal [247]. In addition, the morphology of MAX also affects resistivity, mainly due to different surface areas of MAX with different appearances, with a larger specific surface area providing more surface area. During the contact process between electrons and external electrodes or other materials, it increases the contact points for electron transmission and improves conductivity efficiency [248].

#### Superconductivity

Owing to the strong covalent and ionic bonding interactions, coupled with weaker metallic or van der Waals interactions, this structural characteristic enables electrons to maintain long-range coherence at low temperatures, facilitating the formation of Cooper pairs, thereby promoting the frictionless flow of superconducting current, which is one of the fundamental principles of superconductivity. MAXs exhibit a higher density of electronic states near the Fermi level, which enhances electron–phonon coupling. The d-electron states of elements such as Ti, Mo, and Nb significantly contribute to superconductivity, such as the superconductivity of Mo_2_GaC [249] and Nb_2_SnC [250] which has been demonstrated. The introduction of C or N atoms provides additional electronic states, which promotes the formation of stable electron–phonon coupling systems, thereby improving superconductivity. Experiments have found that certain MAXs exhibit a superconducting transition within a specific low-temperature range, similar to the behavior of traditional superconductors. For instance, the superconducting transition temperature for Mo_2_GaC is 3.7–4.1 K [249], while Nb_2_SC is below 5 K [250]. In addition, Nb_2_SnC, at 7.8 K, exhibits a higher superconducting transition temperature [250].

### Magnetic Properties

By introducing a magnetic element component into M- or A-site, MAXs can realize magnetic properties. Cr_2_GeC is antiferromagnetic [251]; (Cr_1−*x*_Mn_*x*_)_2_GeC formed by partially replacing Cr with Mn induces ferromagnetic polarization. The average magnetic moment and Curie temperature increase with the increase in Mn doping content. The magnetic properties of (Cr_1−*x*_Mn_*x*_)_2_GeC depend on the concentration of Mn and the atomic configuration of Cr and Mn in the crystal lattice [252]. The competition outcome between ferromagnetic and antiferromagnetic states depends on the local chemical composition and the ordered state of the M sites, including (Cr,Mn)_2_AlC [253], (Cr,Mn)_2_GeC [252], (Cr,Mn)_2_GaC [113, 114], (Mo,Mn)_2_GaC [115], (V,Mn)_3_GaC_2_ [162], Cr_2_AlC [67], Cr_2_GeC [239], and Mn_2_GaC [80]. (Mo_2/3_RE_1/3_)_2_AlC, a series of the magnetic i-MAXs, with RE standing for Ce, Pr, Nd, Sm, Gd, Tb, Dy, Ho, Er, Tm, and Lu, exhibit a special microstructure of quasi-two-dimensional magnetically frustrated triangular lattice layers covering the Mo honeycomb structure [42]. The introduction of A-site elements also provides a new pathway for the modulation of magnetic properties [38]. Fe, Ni, Co, and Mn with 3*d* electrons have been generally added into the A-site. V_2_(A_*x*_Sn_1−*x*_)C exhibits hysteresis lines with an "S" shape at low temperatures, and the saturation magnetization intensity gradually decreases with increasing temperature, which indicates that it is a typical soft magnetic material. This strong magnetic modulation that relies on element combinations can precisely control the magnetism of MAXs.

### High-Temperature Oxidation Resistance

**Fig. 8:**
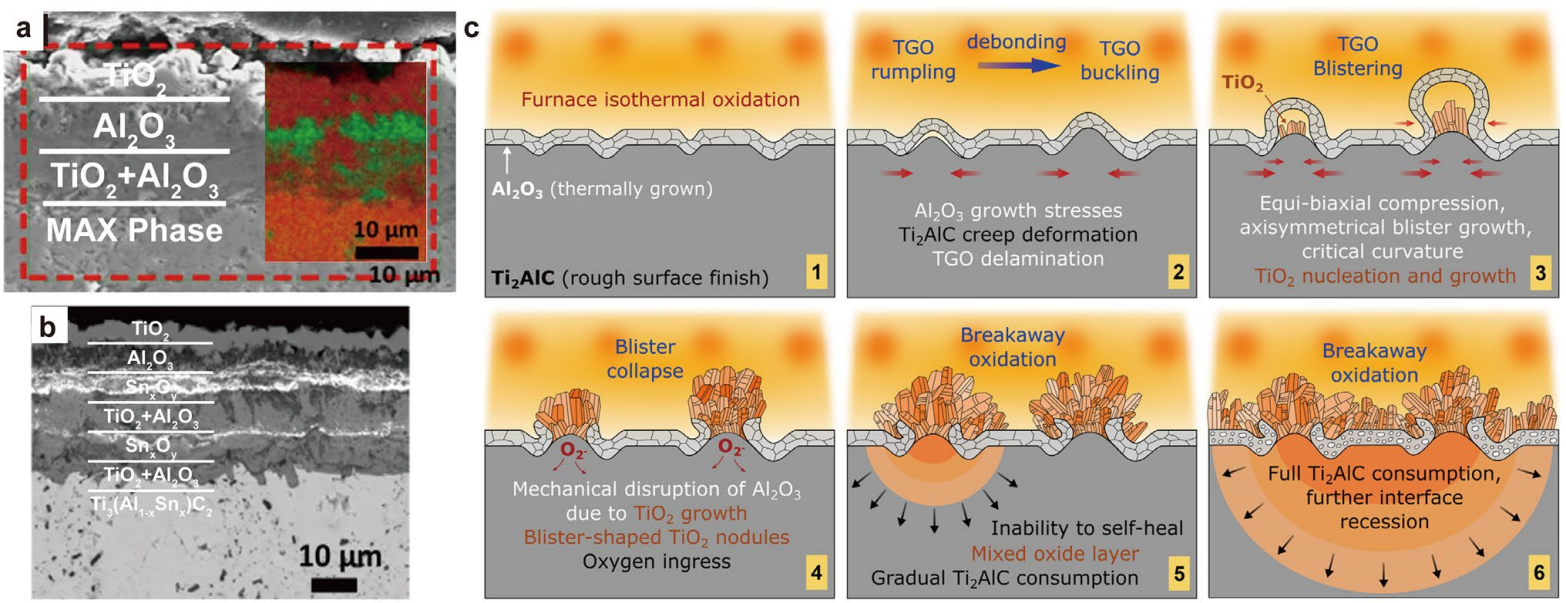
**a** SEM image of the cross section of Ti_3_AlC_2_ after oxidation at 800 °C Reproduced with permission from Ref. [254]. Copyright 2019 The American Ceramic Society. **b** SEM image of Ti_3_Al_0.8_Sn_0.2_C_2_ after oxidation at 850 °C for 30 h. Reproduced with permission from Ref. [254]. Copyright 2019 The American Ceramic Society. **c** Schematic representation of oxide scale rumpling/buckling, blistering, and subsequent breakaway oxidation. Reproduced with permission from Ref. [257]. Copyright 2019 Elsevier Ltd

The oxidation resistance of MAX at high temperatures primarily stems from the diffusion behavior of their specific metal elements, especially those containing Al elements. At high temperatures, the Al atoms tend to diffuse to the surface, forming a dense Al_2_O_3_ protective layer that effectively prevents oxygen penetration (as shown in Fig. 8a) [254]. However, the grain size of MAXs significantly affects the diffusion rate of the Al element. For Ti_2_AlC with small grain size, Al atoms can quickly diffuse to the surface of the grain and uniformly form an Al_2_O_3_ protective layer. On the contrary, for Ti_2_AlC with large grain sizes, Al atoms are difficult to precipitate inside the grain and form a continuous Al_2_O_3_ protective layer, resulting in weaker oxidation resistance. Additionally, when large grain Ti_2_AlC precipitates Al at high temperatures, the matrix does not directly transform into TiC, but instead forms a sandwich structure of Ti_3_AlC_2_ and TiC. This transformation is accompanied by volume contraction, leading to surface cracks that facilitate oxygen infiltration, thus significantly reducing oxidation resistance [255]. Replacing Al with low melting point elements (such as Sn) can lower the temperature of crack healing caused by oxidation, as the oxidation reaction temperature of Sn is lower than that of Al. Specifically, SnO_2_ can form at 460 °C, whereas Al_2_O_3_ requires 900 °C. The high diffusivity and fluidity of Sn facilitate crack repair through oxidation reactions [256]. Nevertheless, these properties can also lead to the diffusion of Sn to the sample surface, promoting the growth of unprotected SnO_2_. Moreover, the small Al/Ti atom's ratio promotes the growth of a non-protective rutile-TiO_2_ scale (as illustrated in Fig. 8b), which in turn affects the alloy's oxidation resistance [254].

Surface roughness also has a significant impact on oxidation resistance. A rough surface increases stress concentration points, making the Al_2_O_3_ protective layer more susceptible to thermal stress during the initial stages of oxidation, resulting in uneven distribution of stress and the phenomenon of rumpling. Wrinkles can exacerbate the accumulation of compressive stress, causing unstable deformation of the oxide layer in these high stress areas, gradually forming a bubble structure. The irregular morphology of the rough surface makes these bubble structures more likely to form. When bubbles burst under external stress or mechanical disturbance, the exposed matrix in the rough area becomes a pathway for oxygen, accelerating the infiltration of oxygen and leading to the formation of a porous mixed oxide layer (as shown in Fig. 8c) [257].

### Corrosion Resistance

The A element can form a stable oxide or nitride protective layer in the corrosive environment, such as aluminum forming an alumina layer and silicon forming a silica layer, which effectively isolates the corrosive medium. In addition, MAXs have a high melting point and excellent thermal stability allowing them to maintain their structural integrity at high temperatures and are not susceptible to thermal decomposition or phase transformation. However, corrosion remains a key factor limiting their long-term use and reliability. In acidic and alkaline environments, MAXs show ceramic material properties with good corrosion resistance, which is mainly related to the elemental composition, whether the M/A element reacts chemically with acid and alkali, and in addition, whether the surface passivation layer can be formed quickly or not, which also determines the corrosion resistance of MAXs in acidic and alkaline environments. It was shown that Ti_3_SiC_2_ is very stable in NaOH, HCl, and H_2_SO_4_ concentrated/dilute solutions with negligible mass loss (< 2 $\mu\text{m}$ yr^−1^) over six months. The corrosion rates in dilute HF and concentrated HNO_3_ were 5 and 13 mm/yr, respectively. However, in the dilute HNO_3_ solution, the corrosion rate was as high as 250–320 mm yr^−1^, which was mainly due to the dissolution of Ti elements into the corrosive medium, leaving behind a Si-rich layer that was oxidized to SiO_2_ in HNO_3_ [258]. Cyclic polarization and chrono-current tests in HCl and H_2_SO_4_ dilute solutions showed that an irreversible electrically insulating layer was generated on the surface of Ti_3_SiC_2_, and this protective film may be responsible for its corrosion resistance. Due to the complex Lewis acid reaction at high temperatures, dissolved A-site elements diffuse into the atomic layer toward the inward molten salt and fluoride salt, resulting in poor corrosion resistance of MAXs in molten salt and fluoride salt environments [259].

## Functional Applications

**Fig. 9:**
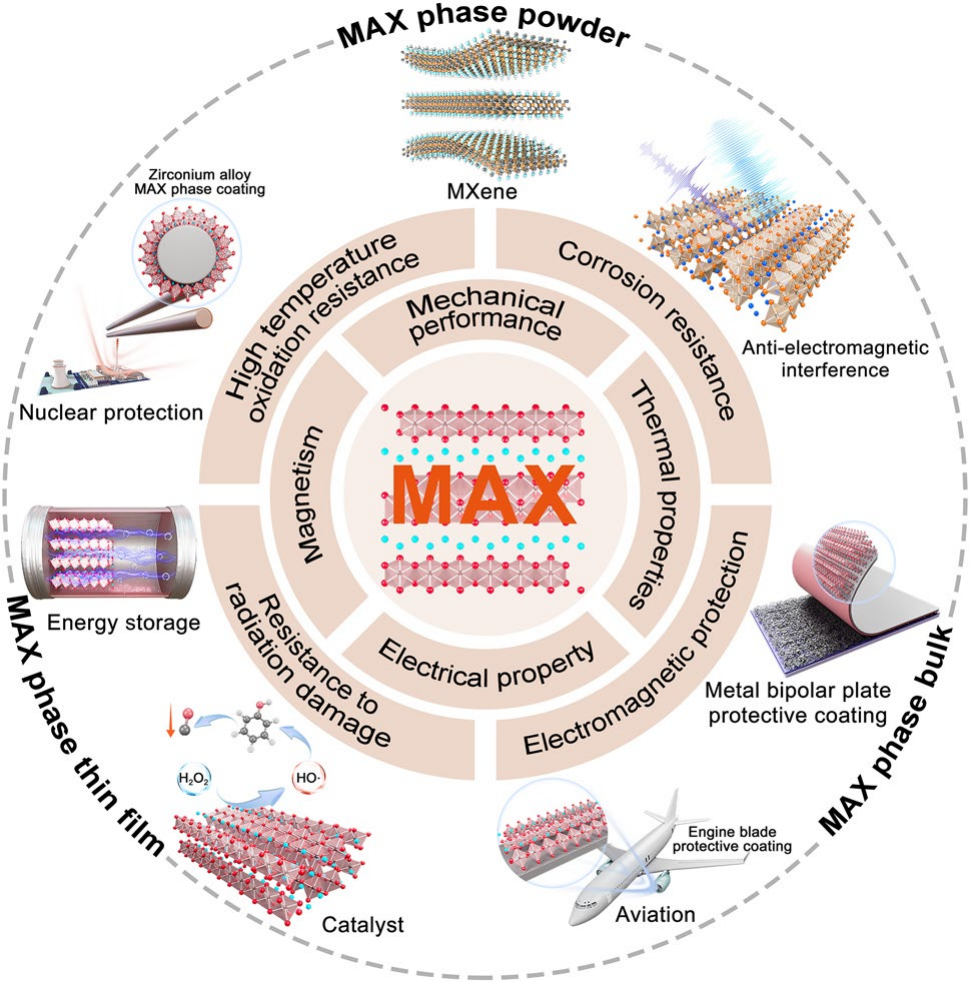
Properties and applications of MAX series materials

MAXs, due to their unique layered structure, combine the advantages of metals and ceramics and have excellent high-temperature resistance, oxidation resistance, thermal shock resistance, mechanical strength, and electrical conductivity, providing support for technological progress and innovation in fields such as aerospace, automotive, electronics, energy, and chemical engineering, as shown in Fig. 9.

### Powders of MAXs

Powder materials exhibit significant advantages in processing flexibility, material performance improvement, rapid reactivity, lightweight and high strength, and microstructure controllability. In addition, the physical and chemical properties of powder MAXs can be regulated and controlled by adjusting the particle size and morphology, thereby improving their functional performance. Therefore, powder MAXs are widely utilized in fields such as electromagnetic shielding and absorption, energy storage and conversion, composite material construction, catalytic reaction regulation, and 3D printing materials.

#### Electromagnetic Interference

Electromagnetic pollution generated by mobile phones, antennas, and security devices also harms human health. Therefore, there is an urgent need to develop high-performance shielding and absorbing materials for electromagnetic protection or electromagnetic compatibility management of electronic components in both military and civilian electromagnetic interference management. MAXs exhibit unique advantages in electromagnetic interference shielding and absorption due to their layered structure, band structure, electronic properties, controlled planar structure, and a wide range of element composition choices.

The EMI performance of MAX powders exhibits significant microstructure dependence [260]. When the particle size of MAX powders decreases, the number of particles per unit volume increases, and the average distance δ between particles decreases, which helps to form a more effective conductive network and promote absorption performance [14]. In addition, as the amount of MAX powders increases, the free electron density and electron transfer efficiency increase, resulting in enhanced dielectric loss, reduced reflection loss, and thus improved absorption performance. Future research focuses on how to realize the geometric configuration design via the structure orientation control technology and composite material construction methods. To understand the electromagnetic interference shielding mechanism of MAX enables the enhancement of EMI performance. In addition, the hollow rod-shaped MAX phase exhibits excellent microwave absorption performance due to its unique microstructure, which facilitates impedance matching and dielectric loss [261].

#### Electrochemical Energy Storage

Based on the fully adjustable physicochemical properties induced by the multi-element composition and layered structure of MAX powder, it exhibits potential as a functional material for electrochemical energy storage electrodes. Due to its layered structure, the large theoretical capacity of A-site atoms, and good conductivity, it was once highly anticipated as an anode material [262]. Still, its performance did not meet expectations. Recent studies have revealed that the layered structure advantage of micron-sized (or larger) MAX particles, coupled with the inability of A-site elements with high specific capacity to function, significantly reduces electrochemical performance. According to theoretical calculations and experimental results, reducing particle size can effectively harness the advantages of the MAX and enhance its energy storage performance [263, 264]. Compared to traditional electrochemical electrode materials, MAX particles exhibit a higher density and stable lattice valence bond relationships, which makes it challenging for electrolyte ions to migrate and transform the valence bonds of MAXs under potential fields. Additionally, MAX particles are predominantly prepared using a top-down method, making it difficult to obtain nanoscale ultrafine particles. This results in the inability of active elements inside the particles to contribute to reaction charges. Therefore, the development of nanoscale MAX powder particle preparation technology is crucial [16, 17].

#### Catalysis

MAX powder materials can provide a larger specific surface area, allowing more constituent metals M to participate in catalytic reactions and promote reaction rates. In addition, the ceramic properties exhibited by MAXs enable them to maintain catalytic activity even in high-temperature and corrosive environments. The rich elemental composition and structure of MAX also provide a foundation for the regulation of catalytic function. The Cr_2_AlC MAX phase as a catalyst has significant advantages in catalyzing wet peroxide oxidation (CWPO), including significantly reducing the generation of carbon monoxide (CO), excellent chemical stability, and reusability [265]. Its unique surface structure and lower metal leaching further enhance the environmental friendliness of the catalyst. MAX catalysts can also improve hydrogen storage performance; adding 7 wt% Ti_3_AlC_2_ to MgH_2_ can lower the dehydrogenation starting temperature to 205 °C [266]. Meanwhile, the apparent activation energy (104.7 kJ mol^−1^) of MgH_2_ sample with 7 wt% Ti_3_AlC_2_ addition was significantly lower than that of the original MgH_2_ sample (50.4 kJ mol^−1^). The high catalytic activity of Ti_3_AlC_2_ is attributed to the ability of H atoms to bind to the interstitial positions of the Ti–Al layer.

#### Composite Material Reinforcing Agents

Based on the synergy of properties and functions, MAX powders are utilized as additives in composite materials to enhance various mechanical properties, including strength, high-temperature resistance, and corrosion resistance. Metal-based composites incorporating MAX powder exhibit not only high strength, modulus, and hardness but also demonstrate excellent machinability, friction, and wear resistance, as well as significant damping capacity. There exists a certain contradiction between the mechanical properties and damping capacity of composite materials [267, 268]. Specifically, while the addition of hard and brittle dispersed particles (such as SiC) can enhance the mechanical properties of composites, it can also pin dislocation movement, thereby affecting damping performance. Therefore, it is proposed that by replacing traditional hard and brittle reinforcements with MAX powders possessing plastic deformation and high toughness, the pinning effect on dislocation movement can be minimized, thus achieving a synergistic enhancement of both strength and damping capacity [269].

#### Precursor of MXene

The geometric structure (particle size, morphology) of MAX powder materials directly affects the preparation method of MXene and the morphology of the obtained MXene materials. In addition, the M-A/M-X bond energy of MAXs also determines the difficulty of MXene etching. The smaller grain size of the MAX usually has a larger specific surface area, which helps accelerate the acid etching reaction and accelerate the synthesis process of MXene. The obtained MXene has richer active sites and a larger specific surface area, which can enable MXene to exhibit higher reaction activity and efficiency in catalysis, sensing, and energy storage [270]. Large-sized MAX grains typically contribute to the formation of a uniform MXene layer structure while maintaining more consistent surface properties and higher electrochemical conductivity [271, 272]. In addition, larger-sized MXene can provide higher mechanical strength [273]. Therefore, regulating the grain/particle size of MAX is key to optimizing the preparation and functionality of MXene. Exploring new MAXs, guiding the control of the geometric structure and valence bond relationships of MAX powder materials, and developing environmentally friendly MXene synthesis methods are the foundation for promoting the commercial application of MXene materials.

Although MAX powder materials have demonstrated potential applications in various fields owing to their unique physical and chemical properties, reducing preparation costs, controlling uniformity, and improving surface stability are serious challenges for large-scale applications. Therefore, developing MAX powder materials with unique geometric shapes, adjusting the surface and interface properties of MAX powder, optimizing the interface bonding strength with the matrix material, and comprehensively improving the functional performance of MAX powder materials are crucial for the application of MAX in specific environments.

### Bulk of MAXs

Bulk materials exhibit a denser overall morphology, typically possessing higher mechanical strength, hardness, and toughness, capable of withstanding greater external impact or compression. Usually, traditional mechanical processing such as cutting, drilling, and forging, can be used to shape and structure them, making it easier to manufacture complex structural components. Due to the continuity of its internal structure, it can form a complete electronic conduction path, usually with good thermal and electrical conductivity, excellent thermal stability, oxidation resistance, and electrical conductivity. In a radiation environment, bulk materials can more effectively resist high-energy radiation (such as neutrons, electrons, ions, X-rays, and gamma rays) due to their dense structure and layered crystal arrangement. These advantages make bulk MAXs widely used in important fields such as mechanical structural components, building materials, electronic components, etc. that require high mechanical strength and stability.

#### High-Temperature Structural Materials

MAX bulk materials exhibit excellent high-temperature stability, oxidation resistance, corrosion resistance, and self-healing properties under high and rapid temperature changes, which are used in the aerospace industry for gas turbine blades, aircraft engine components, and spacecraft insulation layers. MAX can increase the maximum operating temperature by 200 °C [274]. Moreover, MAX shows a good CTE match with standard TBC and thermal growth oxide (TGO) material at high temperatures, which reduces its thermal stress, thereby extending the service life [10, 220]. In the nuclear industry, MAXs are utilized in fourth-generation nuclear reactor components and nuclear fuel cladding materials, owing to their radiation resistance, creep resistance, and self-healing capabilities [275, 276]. The high thermal conductivity and high-temperature resistance of MAX are harnessed in heat exchangers for gas turbine components and solar thermal power generation systems [277]. Furthermore, MAX material serves as a corrosion-resistant reactor liner, high-temperature corrosion-resistant pipeline material, high-temperature furnace lining, and molten metal processing equipment, due to its corrosion resistance, oxidation resistance, and wear resistance at extreme temperatures [13, 278, 279]. The MAX bulk material exhibits high conductivity and a low thermal expansion coefficient, rendering it ideal for high-temperature electrode materials and electromagnetic shielding materials [13, 264, 280]. Leveraging its high-temperature creep resistance, MAX bulk material is also suitable for wear-resistant components of engines and thermal management systems of electric vehicles [281]. As manufacturing technology evolves, MAX bulk materials are poised to play an increasingly significant role in these fields that require high temperature, corrosion, and high strength.

#### Electrical Contact Materials

The primary function of electrical contacts is to establish reliable contact points within the circuit, ensuring efficient current conduction while enduring extreme conditions such as wear, corrosion, and arcing during operation. MAXs are renowned for their exceptional conductivity, wear resistance, corrosion resistance, and high-temperature stability. These attributes render MAXs highly effective in high-current and high-frequency applications, particularly during frequent switching and contact separation processes. They effectively minimize arc and contact point wear, thereby prolonging equipment lifespan. Furthermore, the antioxidant properties of MAXs ensure stable electrical contact performance in harsh environments, such as humidity and corrosive gases [12, 282]. Notably, silver-based electrical contact composite materials containing 10% Ti_3_AlC_2_ (by volume) exhibit performance comparable to commercial AgCdO composite materials [11, 283–285]. Additionally, incorporating Ti_3_AlC_2_ MAX significantly enhances the welding resistance and simplifies the processing of electrical contact materials. The pursuit of non-toxic, high-performance electrical contact materials has emerged as a focal point in this field [286].

#### Connecting Materials

Connecting materials are used to bond two or more components together. MAX bulk materials can provide stronger mechanical properties and thermal stability when connecting complex ceramic, composite materials, and metal components, especially exhibiting significant advantages in high temperatures and harsh environments. By solid-state diffusion, Ti_3_SiC_2_ MAX bulk can bond to Ti_3_AlC_2_ directly [287]. During the bonding process of Ti_3_SiC_2_ and Ti_3_AlC_2_, it was found that Si and Al undergo mutual diffusion, forming a Ti_3_(Si_1−*x*_Al_*x*_)C_2_ solid solution in a pulse current sintering furnace using the rapid current heating method, without the need for any filler compounds or welding agents [288]. It provides a new possibility to seal nuclear fuel cladding tubes onto MAXs.

Despite their excellent mechanical properties, the bulk MAX still exhibits brittleness under certain conditions, particularly in stress concentration points or high-impact environments. While the bulk MAX demonstrates good thermal stability at high temperatures, it still faces oxidation issues in extremely high-temperature environments. Long-term exposure to such environments may lead to performance degradation; therefore, there is an urgent need to improve their antioxidant properties. Additionally, the insufficient interfacial bonding strength between the bulk MAX and other materials during the preparation of composite materials could potentially diminish the mechanical properties of the composites. Furthermore, the bulk MAX encounters difficulties in cutting and forming during processing, especially when manufacturing complex-shaped components, resulting in relatively high production costs and potentially limiting their promotion in certain low-cost applications. To address these issues, future research could concentrate on enhancing the antioxidant properties of the materials, strengthening interfacial bonding, and developing more efficient processing technologies, thereby expanding the application areas of the bulk MAX.

### Film of MAXs

MAX films combine the small particle characteristics of powders and the continuity characteristics of bulks in two dimensions. The self-lubricating, mechanical properties, conductivity, and thermal conductivity make MAX thin films represent the application potential in electronics and electrical engineering. Moreover, MAX thin films exhibit extremely high thermal stability and oxidation resistance under high-temperature conditions, making them highly durable in corrosive and radiation environments. The deposition of MAX on various substrate materials through physical vapor deposition (PVD) and chemical vapor deposition (CVD) has promoted the development of a new generation of high-performance materials.

#### Friction-Reducing Lubrication Coating

Although pure metal coatings are widely used in various industries, their weak atomic bonding forces render them susceptible to wear and corrosion in frictional and chemical environments. Moreover, they tend to oxidize in high temperatures and corrosive media, thereby diminishing their performance. In contrast, MAX thin film materials exhibit exceptional wear resistance and corrosion resistance, owing to their unique layered structure and strong covalent bonding. The M-A-X bonding endows the material with high hardness and friction resistance, while its chemical stability maintains its structural integrity in acidic and alkaline environments. MAXs retain excellent oxidation resistance even at high temperatures. Therefore, when combined with metals, they significantly enhance the wear and corrosion resistance of coatings, offering more reliable protection and extending the service life of coatings under harsh conditions. Jamshidi et al. [289] explored the tribological and corrosion behavior of Al/Ti_3_SiC_2_ composite coatings and discovered that Al-MAX composite coatings exhibit higher corrosion potential and lower corrosion current density compared to pure aluminum coatings. Additionally, the dense oxide film formed by the MAX not only enhances the surface friction reduction performance of the coating but also prevents external material erosion in certain high-temperature extreme environments, significantly broadening the application range and service environment of this type of composite coating.

MAX films are superior to traditional graphite in terms of self-lubricating performance, thermal conductivity, and high-temperature oxidation resistance, making them significantly advantageous as friction lubrication components in extreme environments such as strong acids, strong bases, and high temperatures [290, 291]. Shi et al. [292] studied the tribological behavior of NiAI-Ti_3_SiC_2_-MoS_2_ composite materials and found that MoS_2_ + Ti_3_SiC_2_/NiAl-based composite lubricating materials achieved good synergistic lubrication in a wide temperature range from room temperature to 800 °C. The friction coefficient at 400 °C was only 0.13, and the lubrication effect was supported by a friction film composed of oxide film. MoS_2_ had the main lubrication effect at medium and low temperatures, while the MAX provided a lubrication effect at high temperatures. This type of composite material is expected to perform well in continuous heating environments and is a promising wear-resistant and high-temperature application material. The research results of Zhou et al. [293] show that an increased MAX content can improve the anti-friction performance of composite coatings. In addition, due to the introduction of MAX, the Al_2_O_3_ oxide film generated on the surface of the coating at high temperatures not only improves the surface anti-friction performance but also enhances the high-temperature oxidation resistance of the coating.

#### High-Temperature Protective Coating

High-temperature protective coatings play a crucial role in various fields, including aerospace, energy, chemical, automotive, and electronics. Compared to traditional coatings, MAXs demonstrate exceptional thermal stability and oxidation resistance at elevated temperatures. Additionally, their superior thermal conductivity and self-lubricating properties enable them to effectively reduce friction under extreme operating conditions, thereby significantly enhancing the durability and reliability of the coatings [294, 295]. The composite oxides formed by the oxidation of metal elements in the coating at high temperatures, such as TiO_2_ and Al_2_O_3_, can effectively enhance the bonding strength between the coating and the substrate. Especially after the formation of multi-layer structures, the interface bonding between the coating and the substrate becomes even more compact [2]. MAX coating films serve as a protective coating for refractory alloys and a bonding coating in thermal barrier coatings (TBC) systems. The coefficient of thermal expansion is crucial for reducing stress and avoiding coating peeling. Specifically, the CTE of Cr_2_AlC (12.0–13.3 × 10^–6^ K^−1^) is relatively high, making it suitable for protective layers in metal systems. The thermal expansion coefficients of Ti_2_AlC and Ti_3_AlC_2_ are relatively low, ranging from 8.2 to 9.0 × 10^–6^ K^−1^, and they exhibit better thermal expansion matching with TBC compounds, making them more suitable for use as bonding layers in thermal barrier coatings [274, 296, 297].

#### Nuclear Protective Coating

MAX, with excellent radiation resistance, oxidation resistance, corrosion resistance, strong mechanical properties, and chemical stability, is regarded as potential accident-tolerant fuel (ATF) cladding candidate materials for third-generation light water reactors (LWRs) and future fourth-generation fission devices [275, 298, 299]. The neutron irradiation activity of MAX, including Ti_3_SiC_2_, Ti_3_AlC_2_, and Ti_2_AlC, is comparable to that of SiC materials and is three orders of magnitude lower than that of Alloy 617 nickel-based alloys [300, 301].

V_2_AlC coating exhibits a unique gradient structure along its growth direction. In the region close to the substrate surface, the grains are smaller with more interfaces, whereas in the region farther from the substrate surface, the grains gradually grow larger. This gradient distribution effectively suppresses the excessive aggregation and growth of helium bubbles, thereby enhancing the protective performance of the coating [302]. Ti_3_AlC_2_ and Ti_3_SiC_2_ demonstrate remarkable radiation tolerance upon exposure to high-energy ions like Xe and Kr. Despite being irradiated at high doses, such as 25–30 dpa (displacement per atom), they retain their crystal structure and exhibit rapid self-healing capabilities [303]. Ti_3_AlC_2_ demonstrates a stronger resilience against radiation damage, exhibiting excellent radiation resistance at both low (50 K) and room temperature (300 K) conditions. Although Ti_3_SiC_2_ also exhibits high radiation resistance, it tends to undergo amorphization at higher doses. This amorphization primarily stems from the weaker bonding of Si–C bonds, whereas the Ti–Al and Ti–C bonds in Ti_3_AlC_2_ are more stable, enabling them to withstand radiation damage and recover swiftly. Additionally, both Ti_3_AlC_2_ and Ti_3_SiC_2_ consist of elements with low atomic number (Z), ensuring they do not significantly activate radioactivity under prolonged radiation, which is crucial in nuclear protective materials.

#### Metal Plate Protective Coating

Metal plates are extensively utilized in various fields, such as electrochemistry, corrosion protection, aerospace, and more, owing to their optimized current distribution, enhanced reaction efficiency, and superior corrosion resistance. Introducing coatings can enhance their durability, corrosion resistance, and stability in high-temperature and high-pressure environments, thereby ensuring reliable performance under various extreme conditions. It is crucial to screen coating materials with exceptional corrosion resistance, strength, and stability suitable for extreme environments. Compared to commonly used coating materials such as metals, polymers, and ceramics, MAX films exhibit excellent corrosion resistance, good conductivity thermal conductivity, and flexible machinability. The MAX film coatings on the surface of metal bipolar plates can significantly improve their corrosion resistance and conductivity, presenting considerable application prospects in commercial fuel cells [304]. The MAX film coating exhibits extremely low interfacial contact resistance (ICR) and demonstrates excellent corrosion resistance and durability [305]. In the future, it is necessary to further improve the chemical bonding force and mechanical anchoring effect between the coating and the substrate, such as nitriding or the introduction of transition layers and gradient composite layers. By controlling the changes in composition and structure, gradual transition can be achieved, reducing stress concentration between the coating and the substrate, thereby enhancing the bonding force between the interface, breaking through the interface bonding between MAX coating and substrate materials, constructing integrated electrode materials, and optimizing their functionality.

#### Electrical Contact Coating

Contact materials play a pivotal role in electrical contact materials, directly influencing the operational reliability and service life of equipment. Although pure copper has excellent conductivity and thermal conductivity, its welding resistance is limited. During the surface melting process triggered by arc discharge and Joule heating, the contacts tend to bond, making separation challenging, which in turn compromises the equipment’s disconnection capability. Currently, copper alloys, copper-based composite materials, and copper–ceramic composite materials are widely used as new electrical contact materials, particularly in applications such as pantograph slides, high-voltage switch contacts, and conductive slip rings. MAX films possess strong oxidation resistance, allowing them to maintain performance in high-temperature oxidation environments. These characteristics enable MAXs to provide long-term reliable performance under harsh working conditions. Furthermore, the layered structure endows them with exceptional mechanical strength and toughness, enhancing their durability underwear and impact conditions, particularly suitable for electrical contact applications involving repeated insertion and high-frequency operations. For instance, Ti_2_AlN [306], Ti_3_SiC_2_ [307], and a series of MAX [308] are sputtered on n-type GaN, SiC, or Cu substrates and demonstrate a low ohmic contact resistivity. The deposited MAX film coating serves as an oxygen barrier, preventing potential oxidation, contamination, or the need for any cleaning steps, thereby enhancing the long-term stability of the device. MAX film coating exhibits a higher thermal capacity and a lower thermal conductivity. Under the influence of an arc, the pure metal coating undergoes significant melting and recrystallization, whereas the composite MAX film coating remains largely unaffected, indicating that MAX film coatings have the potential to serve as protective materials for electrical contact surfaces.

In order to better apply MAX film to practice, the advanced synthesis and characterization technology should be applied to achieve the accurate control of the composition, geometric structure, density, uniformity and interface strength of MAX film, so as to improve its functional performance in new energy, sensors, optoelectronic devices, self-healing, and strain response functions.

MAXs have demonstrated significant application potential in multiple fields due to their unique physical and chemical properties. Their conductivity and high surface area make them excellent in lithium-ion batteries and supercapacitors. Their high melting point, excellent mechanical properties, and oxidation resistance make them suitable for high-temperature structural materials, such as aerospace and turbine components. Corrosion resistance and self-healing properties apply to protective coatings and wear-resistant materials. Thoroughly studying the microstructure and performance characteristics of MAXs is key to understanding their structure–activity relationship and driving behavior. Specifically, through in-depth analysis of the crystal structure, defect distribution, interface behavior, and stress–strain relationship of MAXs, the mechanism of performance changes in different environments can be revealed. This not only helps optimize the design of materials, but also guides their performance prediction and reliability evaluation in practical applications. Optimizing the preparation process to achieve mass production and cost control is the key to large-scale applications. By improving synthesis parameters and increasing yield, costs can be reduced and economics can be improved. In the future, MAXs are expected to be widely applied in the fields of energy, aerospace, and environmental protection, promoting the development of related industries. Despite facing challenges, continuous research and technological advancements will enable MAXs to achieve widespread applications soon.

## Conclusions and Perspectives

This review comprehensively explores the development trajectory, elemental composition, crystalline structure, preparation techniques, formation mechanisms and computational simulation advancements, physical and chemical properties, and applications of MAX series materials. It provides a thorough and accessible guide for researchers in the MAX domain to comprehend the latest developments in preparation technologies, structural decipherment, and functional innovation within MAX series materials. MAX series materials still face unresolved challenges that hinder their widespread applications:

1. How to construct a machine learning system to support MAX innovation research?
  Gathering data on the chemical compositions, structures, and both physical and chemical properties of known MAX series materials and merging this with current experimental practices, computational modeling, machine learning, and deep learning can aid scientists and engineers in predicting structure stability and performance under extreme environments. This may minimize the experimental scope, and accelerate the development of MAX series materials through computational insights and empirical validations. By integrating expertise from materials science, computer science, chemistry, and physics, data sample collection is quickly enriched, and the machine learning systems are updated and refactored. The vision is to create an AI-driven autonomous system for MAX creation, incorporating robotics for synthesis and characterization, and AI for interpreting results and suggesting new experiments, thus achieving a fully automated innovation cycle for MAX series materials.

(2) How to address scientific and rational control synthesis of MAX series materials?
  To answer this issue, the exploration of the reaction mechanism is the foundation; in situ characterization may be a key to addressing how to control purity, density, geometrical morphology, and microstructure. In our opinion, the precision preparation should be transformed from solid-phase sintering into molten salt-assisted and vapor deposition. However, to our knowledge, most production enterprises of MAX series materials are using a solid-phase sintering strategy, which makes it difficult to control nucleation and growth processes based on interfacial atomic diffusion by solid interfaces. High temperature and high pressure can accelerate diffusion dynamics; however, it leads an unavoidable energy consumption. The preparation strategies of pressureless, low-temperature sintering based on solid-phase reactions, and cost-effective molten salt processes should be pushed into mass production as soon as possible. Moreover, the vapor deposition technology should be promoted for use in high-end manufacturing, aviation, and military industries, which are not subject to cost control. The synthesis of MAX series materials via aqueous solution reaction is expected.

(3) How to establish an industrial ecosystem for MAX series materials, leading to their practical application?
  Creating an industrial ecosystem for MAX series materials hinges on recognizing and integrating their unique attributes into existing industrial workflows, overcoming challenges in synthesis, property exploitation, and identifying new application domains. In aerospace, MAX series materials can endure extreme temperatures, which are the ideal components in engines and spacecraft. Their resistance to wear and corrosion also suits for protective coatings in space launch vehicles and marine engineering. How to discover the unique characteristics of MAX series materials, the indispensable properties in specific application scenarios can form competitiveness in a variety of functional materials. It is an important option to develop the functional applications of MAX series materials in aerospace and deep-sea exploration. In addition, the balance of function and cost is also a key parameter that limits practical applications.
  As a multi-element material system, how to design the atomic architecture and micro-geometry of MAX series materials is the basis for regulating its properties and functions. The development of new preparation technology is the premise of realizing its large-scale application. Navigating the innovation investigations by cross-disciplinary may unlock a new era of MAX series materials.
